# Whose Burden Is It Anyway? A Comprehensive Proposal to Reshape Food Safety Review by Treating Food as Medicine

**DOI:** 10.1017/amj.2025.10088

**Published:** 2025-12

**Authors:** Katya S. Cronin

**Affiliations:** 1 Fundamentals of Lawyering, The George Washington University Law School, Washington D.C., U.S.; 2 Global Food Institute & Core Faculty, Institute for Food Safety and Nutrition Security, The George Washington University, Washington D.C., U.S.

**Keywords:** food as medicine, food safety, food reform, food policy, GRAS, food additives

## Abstract

With more than sixty percent of U.S. adults struggling with at least one diet-related health condition, the relationship between nutrition and public health has never been clearer. Indeed, for the first time in over a century, food has a prominent place on the national political stage and is one of the exceedingly few issues that has garnered bipartisan support. The recent rise in popularity of “Food Is Medicine” initiatives, which seek to provide medically tailored or healthy meals to vulnerable populations, underscores the critical importance of food to public health. Yet, while “Food Is Medicine” is shifting the insurance, business, and nutritional landscape, the Food and Drug Administration (“FDA”) — the primary regulator in charge of both food and drug safety — treats food as anything but medicine. Known and unknown food additives, color dyes categorically banned in other countries, chemicals leaching from paper and plastic, and environmental toxins and pathogens all contaminate our food and sicken children and adults alike. All the while, the FDA acts as if it were powerless to fulfill its mission of protecting the public from unsafe food.

In a time when the political will to reform our food system and rid it of harmful chemicals and ingredients is high, this article offers a blueprint for how to do so in a scientifically grounded, legally consistent, and lasting manner. Starting from the basic premise that food can be medicine, the article explores ways to bring food safety review closer to the much more demanding safety process for pharmaceuticals. Part I defends the premise that food is every bit as important to human health as medicine — both for good and for ill — and posits that food safety should be regulated just as much as (even if not in identical ways to) the safety of medical drugs. Parts II and III offer a comparison between the rigors of drug safety review, albeit with its own set of problems, and the laxity and at times utter lack of food safety review. Finally, Part IV advances a comprehensive package of both legislative and regulatory reforms, all designed to shift the *de facto* burden of proving the safety of food ingredients from the overtaxed FDA and the overburdened consumers to food manufacturers.

## I. Introduction

The United States is one of the most economically developed nations in the world, yet the health of its citizens is failing. Despite spending the most on health care, Americans “are the sickest and die the youngest compared with nine other high-income nations.”^1^ While this tragic picture can be attributed to a slew of factors — economic, social, and environmental, to name a few^2^ — much of the blame lies at the feet of our diets.^3^ Over half of U.S. adults suffer from chronic diet-related diseases, such as obesity, cardiovascular conditions, irritable bowel syndrome, and cancer,^4^ and diabetes alone affects more than 11.5% of the population.^5^ More than 80,000 new cancer cases a year are linked with suboptimal diet among U.S. adults.^6^ Even maladies not typically associated with food, like chronic kidney disease, are caused to a large degree by nutritional factors.^7^ While diet itself is a complex problem, encompassing malnutrition, nutrient deficiencies, irregular feeding, overconsumption, diet quality, low physical activity, and more,^8^ the chemical load of our food supply is a major, yet often overlooked, culprit.^9^ Food chemicals have been linked to neurological and immunological problems, obesity and cardiovascular disease, endocrine disruptions, impaired brain development, cognitive and behavioral issues such as ADHD, and cancers.^10^

*How did we get here?* For millennia, human food came in whole form — the carrot in the ground, the apple from the tree, the milk from the cow — and was prepared at home.^11^ This was not always an idyllic existence; food in those days rotted easily, carried pathogens, and was contaminated with heavy metals from soil, cookware, and even intentional use.^12^ Synthetic chemicals, however, did not exist and were not a salient issue for food safety until the late nineteenth century.^13^

With the dawn of industrialization, food manufacturers began using synthetic chemical compounds for the purposes of food preservation, shelf stability, flavor and texture enhancement, and nutrient supplementation.^14^ While these developments provided some important benefits, they also introduced new dangers to human health.^15^ Early examples of food additives include stimulants in drinks, formaldehyde in milk, and borax in meat.^16^ Congress responded to this rapid proliferation of chemical food additives that could be injurious to consumers first with the passage of the 1906 Pure Food and Drugs Act,^17^ and later with the 1938 Food, Drug, and Cosmetics Act (“FDCA”).^18^ This early food safety regime placed the burden of proof for food safety on the FDA.^19^ Manufacturers were allowed to introduce substances into the food supply without advanced notice and authorization, and the FDA had to affirmatively prove that these substances were harmful for human consumption before it could deem the relevant food products adulterated and remove them from the market.^20^

The passage of the 1958 Food Additives Amendment ostensibly flipped the burden of proof for intentionally added ingredients onto food manufacturers,^21^ who now had to affirmatively prove that their proposed additive was safe for human consumption before they could receive FDA authorization to include it in food products.^22^ In theory, this burden shift should have ensured a much safer food supply. In practice, however, low statutory safety requirements and outsized exceptions allowed manufacturers to do the bare minimum and still placed a high load on the FDA to chase down and weed out unsafe substances.^23^ In turn, skewed market incentives, a slim workforce, and a de-prioritization of food safety in FDA’s overall agenda meant that the agency was in no position to shoulder this functional burden.^24^ Ultimately, the burden passed on to consumers to painstakingly weed out unhealthy foods or endure the increasing number of chronic illnesses caused by our vulnerable, polluted, and sometimes dangerous food supply.

*Is there a way out*? While food advocates and health care professionals have been sounding the alarm bells on these trends for decades,^25^ these issues have finally become a mainstream topic of discussion.^26^ Nutrition and food safety are increasingly a primary concern for consumers, voters, and politicians alike.^27^ The recent rise of the “Food As/Is Medicine” movement illustrates the heightened awareness about the crucial role food plays in both causing and alleviating illness.^28^ Similarly, the current administration’s focus on “Mak[ing] America Healthy Again” has led HHS Secretary Robert F. Kennedy Jr., FDA Commissioner Marty Makary, and USDA Secretary Brooke Rollins to make a significant push towards addressing dangerous food chemicals and additives.^29^ Working within the existing statutory confines and practical realities of FDA’s limited capacity to police food safety, however, can only go so far. Therefore, despite enormous political will, the current “wins” in this area have come in the form of calling for voluntary commitments from industry^30^ — gains that are neither permanent, nor binding,^31^ and do not solve the safety problems that will inevitably arise with subsequent waves of new chemical additives.

This article offers a blueprint for reshaping food safety review in a more effective, enduring, and consistent way by requiring that food products meet rigorous safety standards akin (though not identical) to those required for pharmaceuticals. The basic premise of the argument is burden-shifting. The proposed reforms seek to realign statutory requirements and regulatory incentives in a way that lifts the burden from sickened consumers and an overworked FDA, and places it onto those who stand to gain the most from the introduction of food chemicals into the food supply — the manufacturers. The article proceeds in four parts and has both a descriptive and a normative component. Part I interrogates the central claim of the recent “Food Is Medicine” movement — that food is, or at least can be, medicine. This part reviews both the historical and contemporary links between food and medical drugs and posits that one implication of viewing food as medicine in terms of efficacy is that it should also be treated as such when assessing its safety. Next, the article offers a comparative analysis of two regulatory regimes. Part II examines the process for approving new drugs and the burden of proof on drug manufacturers to demonstrate safety before they are allowed to market their products to consumers. Part III shifts gears to food regulation and catalogues the many and significant ways in which food safety review lags immensely behind drug safety assessments and falls short of protecting consumers. Finally, Part IV puts forth a comprehensive package of proposed reforms that takes a page from the playbook of drug regulation and seeks to alleviate the administrative burden on the FDA and the health burden on consumers by holding food manufacturers responsible for the safety of their products.

## II. Why Treat Food as Medicine?

The central claim of the Food Is Medicine (“FIM”) movement is simple: food is — or at least can be — medicine.^32^ While this movement has tremendous intuitive appeal, public support, and strong scientific backing,^33^ the discourse surrounding FIM often ignores a crucial link between food and health: food safety. This section examines this link by providing both a historical perspective and a modern nutritional take, and argues that food can only be medicine if we first ensure that, like any other medicine, it is safe.

### A. The Link Between Food and Medicine

For most of human existence, and up until the middle of the nineteenth century, both food and medicine were exclusively sourced from plants, animals, and other natural sources,^34^ and “the boundary between food and drug — and that between dietetics and pharmacology — was rather blurred.”^35^ From the Egyptian Ebers Papyrus to Hippocrates’ *Nutriment*, early medical treatises readily linked proper nutrition with health and prescribed food as medication for various ailments.^36^ Entire systems of medicine, like Chinese traditional medicine and Ayurveda, relied extensively on medicinal foods.^37^ Likewise, Western medical tradition prior to the mid-to-late nineteenth century wholeheartedly adopted the use of food and food-derived compounds to ensure health and treat diseases.^38^ These early traditions, however, were also acutely aware of the dangers that unsafe food could pose.^39^ The knowledge of food toxicity, dose-dependent foods, and food pathogens was carefully passed down to the next generation through oral tradition and, later, scientific notes and records.^40^

The modern-day Food Is Medicine movement embraces the interconnectedness between food and health but often lacks a food safety focus. The movement initially began in response to the HIV/AIDS epidemic in the 1980s, when volunteers brought food to afflicted people in the absence of formalized medical treatment.^41^ In the 1990s, the use of medically tailored meals as a form of medical care increased with the passing of the Ryan White CARES Act.^42^ These efforts eventually led to the creation of the Food Is Medicine Coalition, a group of nonprofits and other community-based organizations dedicated to addressing hunger.^43^ The FIM movement in its present-day form gained nationwide recognition in 2022 after the U.S. Department of Health and Human Services (“HHS”) developed an initiative under the same name.^44^ Today, HHS’s Office of Disease Prevention and Health Promotion coordinates federal and state government efforts to provide clinical care access, food, and educational support to medically vulnerable populations.^45^ These include medically tailored meals, prepared and designed by registered dietitian nutritionists for patients with complex medical conditions; medically tailored groceries for patients with diet-related medical conditions; and produce prescriptions in the form of vouchers or debit cards.^46^ An increasing number of states have also passed FIM legislation that provides coverage for direct food interventions and nutrition education.^47^ FIM interventions not only increase the consumption of fresh and nutritious food, but also significantly reduce chronic disease risk factors and aid healing.^48^

The FIM movement clearly recognizes the role that food plays in health — that an unhealthy diet (usually defined as one rich in sugar, fat, and sodium) can cause diet-related diseases and worsen other medical conditions, whereas a healthy diet (usually defined as fresh fruits and vegetables and clean protein) can have a tremendous medicinal effect.^49^ What the FIM discussion often neglects, however, is that *every* medicine needs to be not only effective but also *safe.* Food — even prescription produce — can be a vector for unsafe agents, such as environmental chemicals and pathogens, and can have detrimental acute or chronic health effects on patients.^50^ Food safety becomes an even more pertinent concern with the proliferation of synthetic chemicals in processed foods coupled with the virtual certainty that, even in the best of circumstances, patients with complex or diet-related medical conditions will rely on processed food for at least part of their diet.^51^

### B. The Link Between Synthetic Pharmaceuticals and Processed Foods

The significant advances in chemistry and manufacturing capabilities during the country’s science and industrial revolutions brought on rapid proliferation of synthetic pharmaceutical agents and processed food products that changed the landscape of both nutrition and medicine.^52^ These developments, however, did not break the link between food and medicine; they provided an additional dimension to it.

*Chemically*, pharmaceuticals and processed foods are closely linked. Indeed, many early pharmaceuticals (analgesics and antipyretics) and early food additives (dyes) originated from the same source — coal tar.^53^ And often, both drugs and food products relied on the same chemical compounds: synthetically derived salicylic acid, for example, was widely used both as pain and fever reducers (and, in its esterified form, later became the active ingredient in Aspirin) and as a common food preservative.^54^ *Functionally*, these powerful compounds held a lot of promise to alleviate various health-related threats. Pharmaceuticals, of course, do so by interacting with the body on a molecular level, while processed foods contribute to public health by staving off food rot, controlling pathogen proliferation, supplementing vital nutrients, and even providing sustenance for populations without access to fresh food.^55^ Conditions such as botulism, scurvy, and neural tube defects are exceedingly rare today largely due to synthetic additives in our food supply.^56^

Both synthetic pharmaceuticals and food additives, however, came with the potential to harm human health.^57^ As a result, the predecessor to the modern FDA — established in 1862 and aptly named the Division of Chemistry (renamed the Bureau of Chemistry in 1901) — examined the safety of these potent chemical compounds in *both* medicine and food.^58^ Harvey Wiley, Chief Chemist of the Bureau at the time and often credited with the passage of the first food safety laws, began testing the health effects of various food additives in human subjects because he firmly “believed that everyone in the country was eating chemically treated foods, that all consumers were receiving daily apothecary doses.”^59^ In other words, as a matter of chemistry, the substances in processed food products were in many ways equivalent to those in drugs. And, although chemicals occurred in lower doses and concentrations in individual food products than in pharmaceuticals, early thinking within the FDA determined that consumers’ ingestion of chemicals in pharmaceuticals and their cumulative exposure to chemicals in food both deserved the same caution.^60^

The landscape of synthetic chemicals and food products has changed significantly over the last 100 years, and the chemical load on consumers today is much higher than in Harvey Wiley’s time. More than 10,000 chemicals are used in processed food currently,^61^ appearing in nearly all products from “junk foods” to “healthy” processed foods like almond milk and gluten free items, to even minimally processed foods like yogurt, meat, and dried fruit.^62^ By latest estimates, between sixty and ninety percent of the typical American diet consists of processed food.^63^ Thousands of these chemicals — including ninety-nine percent of chemicals introduced over the last twenty-five years — have never been subject to meaningful safety review.^64^ Of the small minority of chemicals that have undergone some scrutiny, many have been linked to serious detrimental health effects, “including risk of cancer, developmental harm, and hormone disruption.”^65^ And these numbers only cover the chemicals that are intentionally added to food.^66^ Beyond these, our food supply also carries migrated substances, environmental contaminants, veterinary medicinal products, and pathogens, all of which can wreak havoc on human health.^67^ More than ever before, consumers today are truly receiving “apothecary doses” of multiple chemicals with their every meal. Yet, at a time when our government, medical community, and public proudly announce that “food is medicine,” we treat food as anything but medicine when it comes to ensuring its safety.

The recent advances in science and medical knowledge,^68^ combined with the practical reality of the role processed food plays in our diets, necessitate restructuring our food safety regime to mirror the pharmaceutical safety regime more closely. Indeed, although drugs contain chemicals in higher doses and concentrations, many attributes of food products make food safety review arguably more important. For one, most drugs are used for acute conditions and therefore consumers are likely to be exposed to them only for short periods at a time.^69^ Even drugs for chronic conditions are typically taken once or twice daily, and only after onset, usually in late adulthood.^70^ By contrast, most humans eat three to five times a day, every day for their entire life since infancy,^71^ and most consumers tend to stick with the same types of foods over long periods of time, thus risking slow chronic exposure that is difficult to trace.^72^ Second, medical drugs are prescribed by and taken under the supervision and care of licensed physicians, who are better equipped to understand safe doses, account for interactions with other chemicals, and track adverse reactions from the medication.^73^ By contrast, a lay consumer has neither the knowledge nor training — much less the means — to test for and observe side effects from their food consumption or to consider chemical interactions and cumulative loads.^74^ Finally, most medical drugs are taken out of medical necessity, and therefore even established side effects may be tolerable and the lesser of two evils.^75^ By contrast, no single food is necessary for human thriving or healing, and therefore approving new food chemicals is *never* an emergent matter, nor is a somewhat unsafe product ever a worthwhile health tradeoff.^76^

These differences between food and pharmaceutical intake arguably counsel in favor of *stricter* safety review for food products. This article does not go that far, nor does it advocate for food to undergo the *exact same* strict testing as drugs before going to market. That said, the gross disparity between how the FDA considers drug and food safety is illustrative of a foundational error, which must be remedied if the FDA is serious about protecting and enhancing Americans’ health.

## III. New Drug Safety Review

New drugs go through “a lengthy, detailed, and costly approval process to determine [their] safety and effectiveness” before they are approved for market.^77^ “A typical FDA new drug review involves hundreds of thousands of pages of data, and may require reviewers to rerun data analyses, to query companies for more information, and to closely scrutinize individual trial records.”^78^ While this process is far from perfect and does not lack reasonable detractors,^79^ it is hard to dispute the fact that safety review is an essential — indeed, paramount — component of developing, approving, and marketing new drugs.^80^

Drug sponsors begin this lengthy process in the discovery or preclinical phase, usually conducting several rounds of laboratory, in-vitro, or animal testing to assess whether the product exposes humans to unreasonable risk and justifies commercial development.^81^ In addition to pharmacology, toxicokinetic, and pharmacokinetic studies, the FDA also recommends several types of dose studies (e.g., acute, sub-chronic, and chronic toxicity, repeated-dose toxicity, microdose, etc.), genotoxicity, carcinogenicity, reproductive toxicity, immunotoxicity studies, and combination drug toxicity studies.^82^ While none of these studies are strictly required, industry takes this guidance very seriously in the context of new drug development.^83^ Through this process, the FDA can compel the generation of meaningful, balanced, and unbiased data.^84^ New drug sponsors engage with agency representatives at the very beginning of their research and development process and continue to interact with them and receive guidance on additional information that would support a safety determination throughout the preclinical phase.^85^ Thus, the preclinical phase typically takes at least eighteen months and generates numerous distinct toxicity and safety studies.^86^ “Dozens, hundreds, or even thousands of biologically active agents may be tested in this initial stage of drug development for every drug that receives final approval.”^87^ If any of these tests result in adverse reactions, the FDA requires that sponsors submit a safety report as part of their application.^88^ In this phase, most proposed drugs go through a significant “winnowing” process, due in part to “the fact that it takes, on average, twelve years and costs more than $200 million to develop and obtain approval for a new drug.”^89^

Only if this initial exploration shows promise and raises no significant safety concerns, a drug sponsor can file a formal Investigational New Drug Application (“IND”) with the FDA to proceed with human trials.^90^ The IND substantially concerns safety, rather than efficacy, and requires that the sponsor include all previous test results and a plan of the proposed methods of investigation in humans.^91^ Upon approval of an IND by the FDA and a local institutional review board (“IRB”), sponsors begin clinical trials that test for safety, effectiveness, side effects, unwanted interactions, and consistency.^92^ Phase I of these clinical trials usually involves between twenty and eighty healthy human subjects and is designed to determine the safety, metabolic, and pharmacological profile of the drug and “the side effects associated with increasing doses.”^93^ Sponsors can only move to Phase II efficacy trials if the drug does not reveal “unacceptable toxicity” levels.^94^ Phase II trials, involving larger samples of patients with the targeted condition, mostly focus on drug efficacy.^95^ However, in this phase, a sponsor also seeks to determine “the common short-term side effects and risks associated with the drug” and maximum safe dose of the drug.^96^ Finally, Phase III trials occur in larger sample populations and focus on rarer side effects, drug interactions, and other safety and effectiveness information necessary to evaluate the “benefit-risk relationship of the drug.”^97^

Throughout the clinical trial process, sponsors may only move to a later phase of testing if no safety concerns are raised in the previous phase.^98^ Thus, drug safety is the foundation of new drug approval.^99^ Indeed, “at any time during the clinical trials, a drug sponsor is required to notify the FDA of ‘[a]ny adverse experience associated with the use of the drug that is both serious and unexpected,’” and “the FDA may order a ‘clinical hold’ halting the trials if it determines that safety concerns so warrant.”^100^

Due to these stringent safety requirements, about three-quarters of the drugs that reach clinical trials never make it past this stage.^101^ For the remaining one-quarter of proposed drugs, after the conclusion of all three phases of clinical trials — a process that itself typically takes several years^102^ — the drug sponsor submits a New Drug Application (NDA) to approve the product for U.S. marketing.^103^ An NDA must contain (1) the full investigative reports of product safety and effectiveness, (2) the drug articles used for components, (3) the drug’s full composition, (4) the methods used in manufacturing, processing, and packaging, (5) any samples of the drug and the components used per the request of the Secretary of Health and Human Services (HHS), and (6) the proposed labeling of the product.^104^ The NDA may also contain separate pediatric assessments, if applicable.^105^ A typical NDA “frequently runs in the hundreds of thousands of pages, cataloguing the entire history of the drug’s development and reporting on all the premarketing studies.”^106^ Despite this exhaustive record, during the review of the application, FDA’s Center for Drug Evaluation and Research (CDER) may require that the applicant provide additional drug information or even appear for a hearing before the Secretary of HHS.^107^ The filing of an NDA entails hefty user fees for sponsors — for fiscal year 2025, the rates for an application with and without clinical data were $4,310,002 and $2,155,001, respectively.^108^

Preclinical and clinical testing of new drugs is typically not separated by active and inert ingredients, so sponsors must ensure that the test substances used in preclinical and clinical testing are the exact combination substances they intend to market.^109^ The drug substance consists of all the physical and chemical characteristics of a drug, as well as all the manufacturing information of the substance and the specifications needed to replicate the “identity, strength, quality, and purity” of the substance.^110^ Therefore, the data filed with the NDA should reflect the overall safety, adverse reactions, and interactions of the entire drug, including all its active and inert ingredients.^111^ The FDA decides on a case-by-case basis whether to require further testing to see if any of suspect components are unsafe.^112^

Because no amount of testing could ever rule out all possible side effects and concerns, absolute safety is not an achievable standard.^113^ Additionally, depending on the intended benefits and efficacy of the drug, the FDA may be willing to tolerate a higher degree of safety risk to ensure that critically ill patients receive the care that they need.^114^ In recognition of this reality, the FDA also engages in a robust labeling process. As part of the NDA examination, the drug sponsor must submit proposed labeling for review, which must include warnings, indications, contraindications, dosage recommendations, and interactions.^115^ The FDA then reviews the proposed labeling and engages in sometimes lengthy negotiations with the drug sponsor over adding more thorough information.^116^ The labeling process remains active after market approval, with the manufacturer remaining responsible for updating the labeling as new information about adverse reactions and contraindications becomes available.^117^ For instance, the FDA allows drug manufacturers to change their labeling without approval, if such changes serve to *strengthen* safety warnings and add precautions or adverse reactions.^118^ The FDA can further order updates to the labeling post approval.^119^

After market approval and distribution, manufacturers are required to maintain records and reports such that the Secretary can determine withdrawal or immediate suspension upon a finding of an imminent hazard to the public health.^120^ Sponsors are required to submit annual reports that include significant new information from the previous year affecting the safety, effectiveness, or labeling of the drug product and a description of actions the sponsor has taken or intends to take as a result.^121^ Sponsors are also required to update the drug label to include warnings about clinically significant hazards as soon as there is reasonable evidence of a causal association with the drug, even if a definitive causal relationship has not been established.^122^ The FDA may also require further post-approval clinical trials if new safety information arises after the drug is marketed.^123^ Such information could come in the form of a clinical trial, an adverse event report, a post-approval study, peer-reviewed biomedical literature, data derived from the post-market risk identification and analysis system,^124^ or other available scientific data.^125^ If the FDA determines that there is a safety concern with an approved product, it may require specific risk evaluation and mitigation strategies to ensure safe use.^126^ Failure to comply with the imposed strategies may result in civil or criminal penalties, withdrawal of drug approval, or other enforcement actions.^127^

Beyond ongoing safety testing and post-market reporting, drug manufacturers are also required to use current Good Manufacturing Practices (“GMPs”)^128^ and conduct continual purity testing. Manufacturers must have laboratory controls in place to ensure conformity to identity, strength, quality, and purity standards of components, in-process materials, and final drug products.^129^ Each lot of components, drug product containers, or closures must be tested for contamination and microbiological purity,^130^ while each batch of product must be tested for conformance to final specifications, including the identity and strength of active ingredients and, where applicable, for sterility and absence of objectionable microorganisms.^131^

This approval process is not beyond criticism. Drugs with problematic safety profiles have previously gained FDA approval and have been marketed to the public with detrimental health effects and little to no post-market intervention by the FDA.^132^ The cost and length associated with this approval process also create unnecessary barriers to entry and discourage pharmaceutical innovation for rare diseases, smaller patient populations, or by new market participants.^133^ These and many more concerns are beyond the scope of this article. The point of surveying the regulatory safety requirements for new drugs here is not to raise them as a paragon of regulatory perfection but simply to point out that legislators and regulators alike take the safety of drug chemical formulations that would be ingested by the public seriously.^134^

## IV. Food Safety Review

The process of approving new food ingredients is “much weaker” and ineffectual in ensuring safety than FDA’s oversight over prescription drugs.^135^ Unlike medical drugs, which are tested for safety as a complete formulation, without reference to active, inert, or unintended ingredients, whole food products do not undergo *any* safety review.^136^ Instead, it is only individual ingredients that may require safety approval for specific uses. For ease of conceptualization, this article will review the safety requirements of such food ingredients in three groups: (1) intentionally added food and color additives; (2) expected-to-migrate food contact substances; and (3) unintended environmental contaminants. With every subsequent category, meaningful safety review precipitously declines.

### A. Food And Color Additives

Food and color additives have a safety review process that, at first blush, appears similar to the safety evaluation performed for new drugs and has been described by some as “a rigorous statutory scheme.”^137^ The Food, Drug, and Cosmetics Act requires the FDA to “protect the public health by ensuring that … foods are safe, wholesome, sanitary, and properly labeled,” by assessing the safety of food and color additives.^138^

Food additives refer collectively to “any substance the intended use of which results or may reasonably be expected to result, directly or indirectly, in its becoming a component or otherwise affecting the characteristics of any food.”^139^ Typical food additives include flavors and flavor enhancers, fortifiers and nutrient substitutes, emulsifiers, preservatives, stabilizers, binders, acidulants, anti-caking agents, humectants, conditioners, leavening agents, and sweeteners.^140^ The FDA is granted broad authority to evaluate the safety of any food additive by exempting an ingredient from regulation, promulgating “a food additive regulation governing use of the additive,” either temporarily or permanently, requiring “discontinuation of the use of the additive,” or “any combination thereof.”^141^

Color additives, on the other hand, refer to any “dye, pigment, or other substance made by a process of synthesis or similar artifice” or derived “from a vegetable, animal, mineral, or other source,” which is added to food products for the purpose of “imparting color thereto.”^142^ These are regulated separately from any other food additives under Section 721 of the FDCA.^143^ While the FDA regulates all color additives, it only requires certification for synthetic dyes, lakes, and pigments, sourced from petroleum or coal.^144^ By contrast, color additives derived from natural sources, such as plants, minerals, and insects, are exempt from certifications and manufacturers must only comply with identity and purity specifications.^145^

Congress enacted the Food Additives and Color Additives Amendments “in response to public concern about the increased use of chemicals in foods and food processing.”^146^ As is the case with new drugs, the statute requires that, for both food and color additive certification, the sponsors file a petition with the FDA.^147^ This petition must include the “chemical identity and composition of the … additive, its physical, chemical, and biological properties, and specifications prescribing its component(s) and identifying and limiting the reaction byproducts and other impurities.”^148^ It must also include stability data, proposed amount of use, probable exposure, as well as “full reports of investigation made with respect to the safety of the [food/color] additive.”^149^ The regulations further clarify that the safety reports “ordinarily should include detailed data derived from appropriate animal and other biological experiments in which the methods used and the results obtained are clearly set forth,” and “shall not omit without explanation any data that would influence the evaluation of the safety of the … additive.”^150^ Although no specific tests are required, FDA’s Redbook lists a variety of possible preclinical studies, which somewhat overlap with the types of toxicity studies recommended for preclinical drug investigations.^151^ Based on the application and attached comprehensive safety report, the FDA seeks to evaluate whether the color or food additive would be safe, meaning that “there is convincing evidence that establishes with reasonable certainty that no harm will result from the intended use of the … additive.”^152^

The similarities between drug safety review and food/color additive safety review end there. Differences, on the other hand, abound.

### 1. A Markedly Lower Burden of Proof

The scheme for food and color additives requires a notably lower burden of proof to establish safety for food ingredients than for new drugs. Even a cursory comparison between the food additive regulations (which total eight pages) and the new drug regulations (which clock in at 128 pages) amply demonstrates the significantly laxer safety process for food ingredients.^153^ But the differences run deeper than the page count.

First, unlike new drug regulations, which specifically require a “summary of the pharmacological and toxicological effects” and “pharmacokinetics and biological disposition” of “the drug in animals and, to the extent known, in humans,”^154^ the color and food additive regulations only refer in general terms to any safety information gathered during “appropriate animal and other biological experiments.”^155^ Therefore, although in theory a food or color additive manufacturer *can* conduct many of the same FDA-recommended studies, there is no expectation that they should. Typically, food additive petitions include substantially less data than a new drug application, and their focus is almost exclusively limited to the chemical’s toxicology and toxicity, if that.^156^ Indeed, a 2013 study of all approved food additives in FDA’s database revealed that a shocking 63.9% of them lacked *any* feeding toxicity studies, only 6.7% had reproductive toxicology and teratology data, and *two* had developmental toxicology measurements.^157^ Even looking specifically at chemicals with concern level of II or III — for which the FDA recommends reproductive and developmental toxicity studies — a mere twelve percent of these food additives had such data.^158^

Second, and more significantly, unlike the required three phases of clinical studies in humans for new drugs,^159^ there are no required human safety studies for food or color additives. Instead, the regulations only mention that if “clinical investigations involving human subjects are involved,” the petition should include a statement that each such clinical investigation was “conducted in compliance with the requirements for institutional review … or was not subject to such requirements.”^160^ Indeed, back in 1993, the FDA considered adding a separate chapter to its Redbook to provide guidance to industry on human clinical testing for long-term physiological responses to food or color additives, if preclinical data suggests human subjects will not be put at risk from extended exposure.^161^ However, the agency decided to exclude this chapter from its final publication, further deepening the impression that human studies are not expected for food or color additives. This distinction alone means that food and color additives require years less of safety investigations and likely no data for safety in humans specifically. This is particularly problematic because some food and color additives can cause neuro, cognitive, and behavioral issues in humans that cell or animal-based studies often miss.^162^

And even when food manufacturers do conduct clinical studies for food ingredients, those on balance tend to be much more limited.^163^ “Typically, the sample sizes and objectives of food trials are much smaller and more exploratory than the sample sizes and primary objectives of drug trials” and food trials are primarily focused on substantiating specific labeling claims than assessing safety.^164^ “Food trials do not typically require a safety review by the FDA before the trial can commence,” unless the IRB has specific concerns, and, unlike drug trial sample selection, which is “rigorous,” “[s]ubject selection in food trials is broad and varied, often enrolling healthy persons or those with self-described mild health conditions.”^165^ Unsurprisingly, food trials are also significantly cheaper to conduct, involve fewer placebo and blind presentations, and rely more on “[c]ase studies and anecdotal evidence.”^166^ In sum, what little clinical data is generated in support of a food additive’s safety is hardly sufficient to uncover potential long-term carcinogenic, neurocognitive, autoimmune, or other chronic exposure consequences.

Finally, unlike drugs, which are evaluated as complete products, color and food additives are reviewed for safety only as individual ingredients for a specific intended use. This has several implications. For one, approval of an additive for a particular use does not simply usher in a single product, but hundreds or potentially thousands of food products on the market.^167^ Further, examining the safety of individual ingredients in vacuum prevents an evaluation of adverse interactions with other chemicals in the same food product or across the typical consumer diet.^168^ Nor are reviewers able to properly ascertain the “cocktail effect” of additives and the cumulative exposure to chemicals with common mechanism of toxicity, even though this is an express statutory requirement.^169^ Additionally, although color additives contemplate a dose-dependent approval, the reality of food consumption — which, unlike drugs, is not prescribed by a trained medical professional^170^ — makes any such dosing restrictions illusory.

### 2. Exceptions that Swallowed the Rule

What low-level food safety review is contemplated by the statute, and regulations get further gutted by exceptions that have grown large enough to swallow the rules.

In the food additive context, an exception which makes meaningful review of additive safety nearly impossible goes by the acronym of “GRAS,” or Generally Recognized as Safe. During the Congressional debates on the 1958 Food Additives Amendment, legislators discussed the fact that substances such as salt, pepper, sugar, and vinegar, while technically “food additives,” have been used for a long time, and their safety is so widely recognized, that they should be exempt from regulation.^171^ The Amendment, therefore, granted the FDA authority to exempt these substances from regulation when there was a consensus among “experts qualified to evaluate the safety of substances” that they are GRAS.^172^ This rule served to expedite FDA’s review of all food additives on the market following passage of the Amendment and, at the time, seemed to be a limited exception. For the next decade, manufacturers would routinely request FDA opinion letters, hoping for their sponsored ingredient to be included on the published list of GRAS substances.^173^ Over the next few years, however, the FDA, with the help of the Select Committee on GRAS Substances (“SCOGS”), had managed to review only slightly more than 400 substances.^174^

Eventually, the FDA abandoned its review and instead went on to significantly streamline the process.^175^ First, in 1974, the agency clarified that GRAS status could be obtained either through a scientific determination *or* through a demonstration of “a general recognition of safety” “by experience grounded in the common usage in food for substances used in food before 1958.”^176^ Because *many* substances had been used in food prior to 1958 without any meaningful safety review, this clarification opened a wide door for food manufacturers. Subsequently, in 1997, the FDA issued a proposed rule that established a *voluntary* GRAS notification process, through which manufacturers could, but did not have to, notify the FDA of their own private GRAS determinations.^177^ Under this rule, both the safety assessment and the ultimate GRAS determination would be entirely the responsibility of the manufacturer.^178^ If a manufacturer chose to notify the FDA, the agency could engage and either send a ”No Questions Letter" (a type of soft approval), or a “No Basis Letter” that there is not enough evidence in the notification to establish GRAS status, or acknowledge that the manufacturer has voluntarily withdrawn the notification and cease review.^179^ If the FDA sent a “No Basis Letter," however, nothing prevented the manufacturer from withdrawing the notification and continuing to market the food product with the proposed ingredient.^180^

The rule was finalized in 2016.^181^ Ostensibly, under the new regime, a GRAS determination requires the same scientific rigor as that “required to obtain approval” for a food additive under the petition process, except that the manufacturer need not share the actual data on which their claims are based.^182^ In reality, however, the current regulatory scheme allows a manufacturer to (1) obtain a private determination from a third-party scientific body based on “stale, conflict-ridden, and often unpublished, non-peer-reviewed science,” (2) never make the actual data public, (3) not notify the FDA at all, and (4) proceed to market its product for public consumption.^183^ Proponents of the GRAS rule point out that it is in the manufacturers’ interest to engage with the voluntary notification program and receive a “No Questions Letter" from the FDA. That may well be true for those manufacturers who have no concerns about the safety of their ingredients, but the sheer opportunity to evade review is too tempting for industry and too scary for consumers.

Since the FDA instituted the voluntary notification program, it has taken no steps to re-evaluate the safety of GRAS substances or attempt to determine what and how many self-proclaimed GRAS ingredients exist in our food supply. What we do know is that, for the period between 2000 and 2022, there have been only *ten* new food additive petitions filed with the FDA and only *one* of these was filed after the 2016 rule was finalized — a petition for Vitamin D mushroom powder.^184^ By contrast, 756 GRAS notices were filed through the voluntary notification program for the same period.^185^ In other words, 98.7% of all new substances registered since the turn of the millennium were self-declared safe by the industry, not tested and reviewed by the agency.^186^ The data that these determinations relied on, to the extent available, has been deemed “inadequate” — of all GRAS notices filed until 2013, 61.8% lacked feeding studies and 31.4% lacked any data.^187^ Some of those ingredients have subsequently raised significant safety concerns: BHA, for example, has been classified as “reasonably anticipated to be a human carcinogen” by the National Toxicology Program; BHT may disrupt endocrine function by causing thyroid changes and affect development; green tea extract EGCG may increase risk of cancer; and carrageenan is “associated with the occurrence of intestinal ulcerations and neoplasms,” as well as precancerous lesions.^188^ Yet, these substances still enjoy their undisturbed GRAS status.^189^ Indeed, the FDA has been notoriously slow to revoke GRAS determinations, even with strong evidence of negative health effects — partially hydrogenated oils (also known as trans fats), for example, maintained their GRAS status from 1958 until 2015, and were permitted in foods until 2021, despite strong evidence since at least 1990 that they had negative health effects.^190^

Even more disturbingly, experts theorize that around 1,000 *undeclared* ingredients are masquerading as GRAS substances for the purpose of evading meaningful safety review, and neither the FDA nor consumers have any way of identifying them.^191^ As former FDA Deputy Commissioner for Food Michael Taylor stated, “[w]e simply do not have the information to vouch for the safety of many of these chemicals.”^192^ It seems that the only times undeclared GRAS substances come to the attention of the regulators is when one of those ingredients causes an *acute* health scare for consumers. For example, in 2022, there were nearly 500 unexplained cases of liver toxicity, gastrointestinal pain, gallbladder issues, and other serious medical complications that eventually were linked to a French lentil and leek crumbles product made with tara flour.^193^ Tara flour was neither approved as a food additive nor was there a GRAS notice filed for it.^194^ Instead, the manufacturer had made a private determination of GRAS status based on the ingredient’s use in food products in South America and a single six-week study in rats on general and hepatic toxicity conducted in Peru.^195^ Until the company’s voluntary product recall and identification of tara flour as the culprit ingredient, the FDA was not even aware of this additive’s existence on the U.S. market.^196^ Following this massive health scare, the agency initiated its own GRAS review and concluded — two years after the fact — that there is not enough evidence to deem tara flour as generally recognized as safe and that, therefore, to continue using the ingredient in food products, the manufacturer must file a food additive petition for full review.^197^ Such acute health crises, however, are rare with food ingredients — most additives cause health issues only after a prolonged chronic exposure^198^ — and therefore the hundreds or thousands of undisclosed and untested GRAS additives continue to enter our food supply under the FDA’s radar.

GRAS does not apply to color additives.^199^ Colors, however, along with flavors, spices, and chemical preservatives, can nonetheless evade scrutiny by not being listed on the label. The FDCA generally requires that all ingredients in a food product must be listed on the label by their common name, or else, the FDA must consider the product misbranded.^200^ The statue carves out an exception to this express listing requirement, however, for “spices, flavorings, and colorings,” which can be listed as a group without naming individual ingredients.^201^ While color additives subject to certification must be listed by their proper ingredient name, those that are not subject to certification can simply be designated as “artificial color” or “color added.”^202^ Such group listing, however, makes it impossible for consumers to be on notice about ingredients in their food that they may be sensitive to or that may interact with other chemicals in their diet or medication.^203^ It also makes it difficult to track whether a manufacturer has actually complied with the required FDA certifications or has instead included in their product formulation untested and unregulated ingredients.

A similar problem exists for spices and flavorings. FDA regulations state that flavors only have to be designated as “natural” or “artificial.”^204^ “Natural” flavors can include any essence or extract that starts with a natural product and undergoes a series of complex chemical reactions and may, in its final form, be a mixture of multiple — as many as 250 — undeclared chemicals at a time.^205^ Likewise, artificial flavors, spices, and “chemical preservatives,” can be labeled as such without a specific mention of the exact ingredients.^206^ And an incidental additive originating in the spice or flavor does not need to be listed at all.^207^ These relaxed labeling requirements compound the problem created by GRAS because a manufacturer is not only under no obligation to notify the FDA that they are using an ingredient they deem to be GRAS, but they also do not need to notify *anyone* that the ingredient is in the product in the first place.^208^ In essence, these exceptions create a complete black box for what untested ingredients may be hiding in our food, in what quantities, and with what adverse effect to our health.^209^

### 3. Practical Difficulties in Food Safety Review

The safety review gap between new drugs and new food or color additives grows even wider when considering the practical realities of FDA’s review. Although the FDA has oversized responsibilities for food safety, its food budget pales by comparison to its budget for medical devices and pharmaceuticals.^210^ The budget for the Center for Food Safety and Nutrition — the body within the FDA responsible for food safety — was a mere 1.8% of the FDA’s total budget for 2023, and the entire food budget accounted only for eighteen percent of the pot.^211^ The agency’s food budget is also entirely dependent on Congressional appropriations as only one percent of it comes from nominal user fees for color additives ($0.45 per pound of color additive to be certified with a minimum of $288).^212^ Neither food additive petitions nor GRAS notifications generate any user fees, despite the fact that food additives are an increasingly lucrative market — with an estimated market size of over $23 billion in the U.S. alone and $123.98 billion globally.^213^

By contrast, sixty-six percent of FDA’s Human Drug Program’s $2.166 billion budget for 2022 was made up of user fees (between $2 and 4.5 million *per* application), allowing for meaningful engagement with drug sponsors and robust safety review.^214^ These stark differences in disposable funds result in a much thinner and more overwhelmed food safety workforce, one which often must triage and can focus only on the most urgent matters, such as pathogen outbreaks. Unsurprisingly, then, while new drug review (including study follow-ups and hearings^215^) takes three to ten years to complete and involves numerous engagements between drug sponsors and FDA reviewers, a typical food additive review takes less than twenty-four months and relies on much more cursory interactions.^216^

### 4. Lack of Post-Market Action

Finally, significant differences exist in the FDA’s post market review for approved drugs and approved food or color additives. Whereas drug manufacturers have annual reporting obligations, requirements to report any adverse reactions, and a duty to update their labeling for new contraindications — all while facing the looming threat of an FDA-imposed hold or approval cancelation if any source puts the drug’s safety in question^217^ — food and color manufacturers have no general post-approval reporting obligations.^218^ The only reporting obligation imposed on food manufacturers is if they learn of “a reasonable probability that the use of, or exposure to, such article of food will cause serious adverse health consequences or death to humans or animals”^219^ — an acute situation that typically does not arises in the context of food additives.

Even when the FDA is made aware of safety concerns, it has been notoriously slow in using its post-market authority to revoke food and color additive approvals. The most recent example of Red Dye 3 is instructive. The FDA approved Red Dye 3 in 1969.^220^ Due to mounting evidence of safety concerns throughout the 1980s, including thyroid tumors in rats, the agency banned the use of Red Dye 3 in cosmetics in 1990.^221^ Yet, it took an additional *thirty-five* years before it finally revoked its approval of the color additive in use in food products in January 2025, to go into effect in January 2027.^222^ Likewise, the FDA took over *fifty-four* years to revoke its authorization for the use of brominated vegetable oil (BVO) as a food additive.^223^ In 1970, after significant evidence surfaced that BVO could harm vital organs, such as the liver and heart, the FDA downgraded the ingredient from a generally recognized as safe status to that of an approved food additive.^224^ Despite strong scientific evidence, including studies supporting an analogous ban enacted in the UK in the 1970s, the FDA revoked its own approval only in July 2024 — and only after industry had voluntarily stopped using the ingredient for years.^225^

These examples might sound extreme, but sadly, they are representative of the FDA’s slow response to food safety concerns.

Currently, the FDA’s color additives inventory lists nine approved main synthetic color additives (not counting the now-banned Red Dye 3 but including titanium dioxide).^226^ Of these, at least seven have been associated with negative health effects in humans.^227^ In general, consuming artificial food colorings has been proven to increase the amount of manganese and worsening ADHD symptoms in susceptible individuals.^228^ Further, Yellow No. 5 may be linked with negative neurobehavioral changes in children, and several microbiological and rodent studies of Yellow 5 were positive for genotoxicity.^229^ The FDA’s “acceptable” level for Yellow 5 is set at more than sixty times higher than the level that researchers have identified as triggering neurobehavioral effects in a double-blinded, placebo controlled, study of young children.^230^ Blue No. 1 and its analogs have been shown to affect neurodevelopment and hyperactive behavior.^231^ Blue 1 has also been shown to cross the blood-brain barrier even in adults.^232^ Three dyes (Red 40, Yellow 5, and Yellow 6) have been found to be contaminated with benzidine or other carcinogens.^233^ At least four dyes (Blue 1, Red 40, Yellow 5, and Yellow 6) cause hypersensitivity reactions.^234^ Red Dye 40 is also associated with allergic reactions.^235^ Titanium dioxide, first approved by the FDA as a color additive in 1966 on the belief that it does not accumulate in the body, is now known to build up in tissues and to promote precancerous changes and alter the absorption of nutrients from food.^236^ Toxicity tests on Citrus Red 2 and Orange B — two dyes that the FDA recently indicated it would reexamine^237^ — likewise suggest general safety concerns.^238^ Alarmingly, the effects of these dyes are even more pronounced in conjunction with other approved food dyes — a scenario that most closely replicates real-world consumption patterns.^239^ Consumption of over-the-counter medications and vitamins, which likewise include food dyes, further increases exposure to these substances.^240^

Despite many of these ingredients being banned or reexamined in European and other countries,^241^ they are perfectly legal for use in food products in the United States, including for products specifically marketed to children. Indeed, while their consumption here has increased more than fivefold since the 1950s,^242^ the FDA has taken almost no actions to re-examine their approvals. In fact, Red Dye 3 is the *only* color additive that the FDA has banned for use in food products since 1976.^243^

Likewise, a staggering number of food additives remain approved for use in the U.S. despite significant concerns about their safety. While the exact number is hard to determine due to overlap between the various databases, currently, there are approximately 4,000 food additives approved for use in the United States.^244^ Nearly 1,000 of these are not allowed for use in food in the European Union and other developed countries, yet each of these are found in hundreds to several thousand different U.S. food products.^245^

Several categories of food additives have raised significant safety concerns in the scientific community. Nitrates and nitrites, for example, have long been criticized in the U.S. for use as preservatives in meats and other animal products, due to their significant gastrointestinal impact, carcinogenic potential, and thyroid blocking functions.^246^ Emulsifiers directly affect the intestinal microbiome and increase bacterial genes’ pro-inflammatory activity.^247^ Individual additives have also raised significant safety concerns. Sucralose, for example, has been proven to promote inflammation.^248^ Maltodextrins can lead to increased susceptibility to intestinal damage.^249^ Trichloroethylene was banned by the Environmental Protection Agency in December 2024 as “an extremely toxic chemical known to cause liver cancer, kidney cancer, and non-Hodgkin’s lymphoma.”^250^ Potassium bromate was banned in the European Union, the UK, Canada, India, and Peru *and* has been classified as a “genotoxic carcinogen” by a joint committee of the United Nations and the World Health Organization.^251^ Yet, these ingredients continue to be widely used in food in the U.S.^252^ Indeed, in the past twenty years, the FDA has only revoked eight additive approvals: bromated vegetable oil plus a batch of seven synthetic flavoring substances, one of which had long been abandoned by industry.^253^

This dynamic highlights the much lower safety threshold that food manufacturers are expected to cross before they can freely market their chemical compounds to consumers and the much higher safety burden that consumers bear each time they purchase a candy bar.

### B. Food Contact Substances

The burden of proof on manufacturers decreases, and the safety burden on consumers increases even further with the safety review contemplated for food contact substances. Food contact substances are defined as any substances “intended for use as a component of materials used in manufacturing, packing, packaging, transporting, or holding food if such use is not intended to have any technical effect in such food.”^254^ Under the Food Additives Amendment, food contact substances (“FCS”) were considered “indirect food additives” and were regulated in the same way as direct food additives, through a food additive petition.^255^ An exception was created under the FDA’s Threshold of Regulation rules for any substance with a minimal daily exposure level, which does not present any other health or safety concerns.^256^

A third, more expeditious and direct approval mechanism was introduced in 1997 (effective in 2000) by the Food and Drug Administration Modernization Act.^257^ The Act specifically carved out food contact substances as a separate group of additives.^258^ It also created the Food Contact Substance Notification Program, under which an FCS manufacturer could submit a food contact notification (“FCN”) to the FDA for each new additive and, if the FDA does not object within 120 days, the substance gains automatic market approval.^259^ Due to its lower requirements and more predictable timeline, the FCN program is the preferred route of FCS approval for both manufacturers and the agency. Under this abbreviated safety review, the FDA nonetheless claims to conduct rigorous review of the scientific data and to only approve a substance if “new scientific information” demonstrates that the substance has a “reasonable certainty of safety” for the intended use^260^ — in other words, that there is a “reasonable certainty in the minds of competent scientists that a substance is not harmful under the intended conditions of use.”^261^

The reality is different. Because a filed FCN automatically effectuates within 120 days unless the FDA requests more data or otherwise objects, the FDA’s understaffed workforce matters even more than in traditional food additives review.^262^ Moreover, the level of data required for an FCN “depends on the estimated daily intake of the [substance],” an estimate that the *sponsor* determines on their own.^263^ Indeed, a self-determination of “no migration” completely eliminates the need for filing a notice because, technically, the substance does not fall within the definition of a “food additive.”^264^ Given that different testing conditions can produce vastly different levels of migration from food contact materials to food, it is not surprising that most manufacturers greatly underestimate the actual exposure of consumers to their proposed substances.^265^

The negative effects of these (already low) statutory requirements for FCNs are compounded by the same practical realities that weaken food and color additive review in general. Food contact substance notifications come with no user fees and often involve very little, if any, meaningful engagement between sponsors and overburdened FDA reviewers — a feature that has been generously termed “one of the most efficient notification processes within the FDA.”^266^ Similarly to the direct food additive context, here too, GRAS presents an oversized loophole that allows manufacturers to introduce food contact substances into their products on the basis of self-certifications and pinky promises, without any meaningful review.^267^ Indeed, many “[l]eading food and drug attorneys long have advised against seeking the agency’s sanction when marketing without it is equally legal.”^268^ Furthermore, post market review and enforcement is virtually nonexistent for food contact substances.^269^ The FDA has gone decades without rescinding FCN status despite mounting evidence of negative health consequences, ordinarily reclassifying FCNs as ineffective only *after* manufacturers voluntarily phase out the concerning substances.^270^

The low safety burden for approving FCS may not sound particularly troubling when thinking about these substances in the abstract because of the general (wholly inaccurate)^271^ expectation that estimated consumer exposure from migration between food contact materials and foods must be miniscule. The issue becomes much more problematic when examining the outsized detrimental impact on human health of specific FCSs, such as phthalates, parabens, bisphenols (like BPA), perfluorinated compounds (like PFAS), and perchlorate.^272^ Each of these categories of substances has been proven to migrate in significant quantities from food contact materials, causing serious negative health effects.^273^ BPA, for example, is linked with endocrine disruptions, reduced fertility, altered puberty timing, and development of neoplasia; yet, while currently banned for use in infant feeding bottles, it is widely used as a permitted ingredient in plastic bottles and containers and as a polymeric resin to prevent metal corrosion.^274^ Similarly, Perchlorate, which has been linked to major thyroid disruptions, is used as an antistatic agent for plastic packaging and as a cleaning solution in food manufacturing.^275^

Many of these FCS exist in functional groups of thousands of chemicals, all of which have the same or substantially similar effects. The FDA, however, currently regulates FCSs as individual compounds only.^276^ In other words, even if the FDA were to go through the laborious exercise of reviewing and ultimately rescinding an effective FCN — which has not happened in decades — that decision would only apply to a single chemical formulation, allowing a manufacturer to immediately file an FCN for any of the related substances with the same or substantially similar effects. This game of whack-a-mole effectively negates the FDA’s capacity to conduct meaningful post-market review, leading to substances *proven* to cause health issues for consumers to be used and marketed with impunity. PFAS, for example, are a group of tens of thousands of chemicals, yet only a handful of these have been voluntarily phased out of select food contact materials (mostly, grease-proof paper).^277^ Thousands of other PFAS remain widely in use in food contact materials like cookware and HDPE plastic containers despite alarming evidence of their link to a panoply of cancers.^278^ Phthalates are another functional group containing many different substances linked to testicular toxicity, childhood obesity, insulin resistance, arrhythmia, and general inflammation.^279^ While some of those compounds are getting phased out of food products, others — like diisodecyl and diisononylphthalate — are increasingly prevalent in both products and humans.^280^

### C. Environmental Contaminants

Environmental contaminants come in many shapes and forms — pesticide chemicals, legacy chemicals banned for use but persisting in the environment, heavy metals, microplastics, pathogens, and mycotoxins, to name a few.^281^ The problems with environmental contaminants in food are plentiful and could be the subject of several law review articles. Because this area involves joint jurisdiction with both the United States Department of Agriculture and the EPA, FDA’s authority to regulate environmental contaminants in food is significantly curtailed.^282^ In essence, the FDA attempts to regulate environmental contaminants in two ways: (1) by requiring Good Manufacturing Practices to prevent unintentionally added contaminants; and (2) by regulating poisonous and deleterious substances that are not food additives.

The Food Safety Modernization Act of 2011 (the “FSMA”) instituted multiple safety requirements for food manufacturing facilities that aimed to minimize the risk of production contamination.^283^ The Act also augmented the FDA’s enforcement authorities in case of noncompliance, including providing for mandatory recalls,^284^ administrative detention,^285^ and import certification requirements.^286^ Finally, the FSMA also required the FDA to increase its rate of inspections, imposing a minimum inspection frequency of three and five years for high-risk facilities and non-high risk facilities respectively.^287^ However, the FDA’s finalized rule implementing the FSMA’s requirements — originally set to go into effect in January 2026 — has been postponed to mid-2028 due to industry pressure.^288^ It therefore remains to be seen whether the FDA would in fact use its authority to recall products from noncompliant manufacturers.

For poisonous or deleterious substances that are not a food additive and not a pesticide (regulated separately largely under EPA authority), the FDA in turn has the power to: (1) declare a product adulterated, if the substance can be avoided through good manufacturing practices, or (2) set a tolerance, regulatory limit, or an action level, if contamination with the substance is unavoidable.^289^ In theory, if a tolerance, regulatory limit, or action level is exceeded, the product qualifies as adulterated, and the FDA has the right to recall or seize it.^290^ The agency has set tolerances for a handful of contaminants, including nitrites and benzene in bottled water^291^ and aflatoxins in food.^292^ However, the FDA has not set specific levels for most environmental toxins of concern — including PCBs, PFAS, dioxins, perchlorate, radionuclides, and microplastics.^293^ Instead, its entire engagement is monitoring consumer exposure through the Total Diet Study and targeted field tests.^294^ The FDA publishes this data but, other than issuing occasional import alerts that permit (but do not require) the detention of a good without inspection,^295^ it does not otherwise act on the information.

The most involvement the agency has with this type of contamination is with pathogens and heavy metals. For pathogens, the agency takes a strong stance towards Listeria monocytogenes in Ready-to-Eat Foods.^296^ For heavy metals, the FDA established the Closer-to-Zero initiative in 2021, through which it is currently finalizing action levels for lead, arsenic, cadmium, and mercury.^297^ In the meantime, however, recalls for pathogen outbreaks abound;^298^ institutions like Cleveland Clinic note that “[h]eavy metals like arsenic, lead, mercury and cadmium *routinely* taint baby food,”^299^ and consumers are currently suing Amazon over “alarmingly high” levels of toxic metals found in their food products.^300^

## V. A Comprehensive Food Safety Reform

Consumers, advocates, and policymakers increasingly recognize that our food system is broken, polluted, and deleterious to public health,^301^ creating strong momentum for change.^302^ Proposed solutions abound.^303^ What this article adds to the conversation is a comprehensive, concrete, and complete blueprint for how to strengthen food safety in a systematic, predictable, and science-based way. It does so by categorizing existing proposals, adding novel suggestions, and examining the pathways and authority necessary to achieve an effective food safety system overhaul in the current political climate at both the congressional and agency levels. Mindful of the economic, logistical, and administrative hurdles that stand in the way of the agency, the proposals aim to realign incentives and flip both the legal and practical burden of proving safety at all stages of the process from an overworked and understaffed agency and a sickened public to the food manufacturers.

### A. Congressional Actions

A legislative reform that strengthens the current food safety requirements and brings them more in line with the process for approving new drugs offers a direct route to robust and effective food safety review. Ordinarily, proposing a legislative solution to a pressing problem is a near-illusory approach to a crisis because passing legislation in general, and doing so in an expeditious manner in particular, has been exceedingly difficult and unlikely in recent decades.^304^ However, for the first time in nearly a century, political circumstances make meaningful legislative reform possible (and, indeed, likely). Not only are the House, Senate, and White House politically aligned with each other, but they are also aligned with the Make America Healthy Again movement, which aims to radically remake food safety review and to ensure a cleaner food supply.^305^ Indeed, FDA Commissioner Marty Makary has previously signaled that the Administration is “exploring every tool in the toolbox,” including potential “statutory and regulatory changes.”^306^ Beyond that, these goals also receive significant bipartisan support at both the federal and state level and are strongly favored by a majority of voters, with many states pursuing localized reforms.^307^ Harnessing the power of this political and public support and directing it towards meaningful, effective, and permanent reform, therefore, is critical. This section outlines a packet of legislative actions that could accomplish these goals.

#### 1. Close the GRAS Loophole

The most widely supported and impactful legislative change would be to close what many call the GRAS “loophole” to food safety review.^308^ This can happen in several ways.

First, Congress can scrap GRAS altogether. Following the logic of the current GRAS exception for food additives, the FDCA originally offered GRAS status for all drugs that had been on the market prior to 1938 and had retained their exact formulation.^309^ By 1962, however, Congress realized that drugs should not simply be grandfathered in and should instead be subject to rigorous review. Thus, it amended the FDCA to require that all drugs must be proven both safe and effective prior to being marketed.^310^ The FDA created the Drug Efficacy Study Implementation to review the safety of all drugs introduced on the market between 1938 and 1962.^311^ And, while some early drugs were still grandfathered in, at no point after 1962 were drug manufacturers ever allowed to self-certify their products as safe, let alone to do so without even notifying the FDA.^312^ The lack of such an exception did not lead to the sky falling for the pharmaceutical industry;^313^ it merely allowed the FDA, based on actual clinical evidence, to determine whether a pharmaceutical compound is safe for human consumption.

The same should be true for food. The mistaken legislative assumption that food additives in use prior to 1958 were generally safe has now been proven categorically false.^314^ Many of the chemical substances synthesized in the 1940s and 1950s are the very scourges that the EPA, USDA, and FDA now understand cause significant adverse health effects.^315^ Likewise, the assumption that food additives occur in low doses and concentrations and are therefore innocuous is demonstrably untrue.^316^ We know that these substances lead to low-dose chronic exposure that, years or decades down the line, manifests in a variety of chronic illnesses.^317^ Many of these chemicals also can bioaccumulate in human tissues — once ingested, they never leave the body and multiply the negative health effects with every subsequent exposure.^318^ The rapid proliferation of food additives in processed food has also led to the same type of information asymmetry between consumers and food producers that exists in the pharmaceutical space, necessitating closer FDA scrutiny and a more robust information-gathering function.^319^ A categorical removal of the GRAS exception therefore is supported by both logic and science.

Second, if Congress is not inclined to end the GRAS exception, it should honor the original purpose behind it by making GRAS applicable only to substances that have a proven long-term and safe history of use (minimum twenty years) and precluding its application to new substances.^320^ For substances that currently have GRAS status and are already recorded in the FDA’s database — 1,234 substances, as of the date of this writing^321^ — Congress should order a *comprehensive* safety reevaluation by the FDA within five years,^322^ akin to the reevaluation it ordered for pesticides under the Food Quality Protection Act.^323^ For any future GRAS notifications, Congress should mandate that they be accompanied by *all* applicable and available safety data, as is true for new drug applications,^324^ to allow for agency safety assessment or, at a minimum, “judicial and public oversight.”^325^ For these more robust future filings, the FDA should be required to conduct only a *focused* review to (1) verify that there is evidence of long-term use of the ingredient, and (2) do a literature review for any reported adverse reactions or negative safety determinations.

At a bare minimum, Congress must unequivocally end the voluntary notification program and instead mandate that *all* GRAS certifications be logged in with the agency *before* a manufacturer begins to use an ingredient in their food products.^326^ For any currently undeclared substances that have not been logged through the notification system and enjoy under-the-radar GRAS status, Congress should mandate that manufacturers must file a notice, accompanied by evidence of long-term use and all safety data — akin to an IND^327^ — within one year of the effective date. Because these self-certified substances should at least in theory have gone through internal safety review, manufacturers should already have all safety data on hand to file and need time only to put together their paperwork.^328^ In the (likely) event that manufacturers do not have the necessary safety data within that first year, this provision would effectively make the use of such unauthorized and unexamined substances illegal and their products adulterated, unless and until manufacturers decide to file a fully supported GRAS notice or a food additive petition.^329^

Logistically, of course, such changes would pose some difficulty for food manufacturers who have relied heavily on GRAS self-certifications.^330^ They would also temporarily burden the FDA because every single food chemical in use in the food supply would need to be logged in with comprehensive safety information.^331^ This added burden is not a sufficient reason to shy away from reform. First, as a principled matter, the purpose of the FDA is to ensure food safety, not to help manufacturers more easily get a product to market or to maintain a light workload.^332^ Indeed, several former FDA commissioners in charge of food safety have lamented the fact that the FDA currently does not have the authority to fully investigate the safety of food substances.^333^ Jim Jones, the former Deputy Commissioner of the FDA’s Human Foods Program, for example, testified before a Senate committee in December 2025 that “[w]e are several decades behind Europeans and our Canadian counterparts because they have legal mandates to reevaluate chemicals that have been authorized at some point in the past.”^334^ Many of the additional actions proposed in this package — such as user fees, mandatory purity testing, and mandatory post-market reporting — would also significantly lighten the load for the FDA and would instead shift the work onto the food manufacturers. In addition, neither Congress nor the FDA are new to this issue and have previously aptly utilized phasing out periods, tiered review, and graduated tolerances to allow the market the time to adjust to stricter regulation.^335^ Perhaps most importantly, as a practical matter, because GRAS self-certification does not exist in other more health-conscious markets like the EU and the UK,^336^ food manufacturers have either already conducted safety studies for these jurisdictions or have readily available safer alternative ingredients that would allow them to reformulate their U.S.-based products within a reasonable time frame.^337^

#### 2. Repeal the Food Contact Notification Program

In a similar vein, Congress should also repeal the Food Contact Notification Program.^338^ As discussed earlier, this Program was instituted for the purpose of efficiency, but its efficiency has now vastly overtaken its efficacy. The automatic “approval” of a food contact notice, combined with the strain on FDA resources virtually guarantees that any filed notice would get “approved” and that no amount of evidence would be sufficient for the FDA to revoke it post-market.^339^ Given evidence of the massive detrimental effect of food contact substances like BPA, PFAS, and phthalates on human health,^340^ it is imperative that the FDA re-review the safety of all previously noticed substances and comprehensively evaluate the safety of any new proposed substances through a full petition process.^341^ In this context too, Congress should clarify that no food contact substances could be self-certified as GRAS. Any added burden on the FDA through these actions should be mitigated through the same phase-out periods and graduated review expectations outlined for GRAS notices above.

Relatedly, Congress should also clarify the language of the so-called Delaney Clause. Named after Representative James Delaney, this clause was introduced with the 1958 Food Additive Amendment and provides that “[n]o additive shall be deemed to be safe if it is found to induce cancer when ingested by man or animal.”^342^ Read literally, the provision empowers the FDA to reject any food or color additive if there is *any* evidence of carcinogenicity.^343^ Despite this sweeping language evincing a seemingly zero-tolerance policy towards carcinogens in food,^344^ the Delaney Clause is “surely the most discussed, but perhaps the least used, provision of the FD&C Act.”^345^ Subsequent court decisions have significantly curtailed the applicability of the clause and the FDA’s own reticence to use the clause has rendered it virtually illusory.^346^ One uncertainty about the Delaney Clause is whether it applies to GRAS substances. As written, the provision applies only to “food additives” and GRAS substances are exempt from the regulatory definition of “food additives.”^347^ Congress should step in to clarify that, to the extent the GRAS exception remains in any form, the Delaney Clause should apply to such GRAS substances with the same force as it applies to direct and indirect food additives and color additives.^348^ Congress should also resolve the debate as to whether a *de minimis* exception to the Delaney Clause exists, particularly for food contact substances.^349^ A *de minimis* exception sounds eminently reasonable at first blush given the low concentrations and seemingly low levels of migration for many food contact substances.^350^ However, such an exception has proven problematic considering both the chronic exposure to these substances across products and the compounding effect of all food additives in our diet.^351^

#### 3. Institute User Fees for All Safety Assessments

A natural corollary to requiring the FDA to both reevaluate over a thousand currently permitted additives and conduct more thorough pre-market safety review is a proposal that the FDA institute user fees for all food safety petitions — including direct and indirect food additive petitions, food contact notices, GRAS notifications, and color additive petitions (the last of which currently have only nominal fees). This idea has been explored sporadically in the past: a 1993 Report by the Government Accountability Office, for example, suggested user fees as a potential strategy to regulate novel ingredients and technologies.^352^ The idea has a particular appeal today, however, considering the financial and staffing realities at the FDA and across the federal government. The FDA has recently been sharply criticized over its “inability to fulfill critical food safety activities related to its pre- and post-market review of substances added to food.”^353^ Even more recently, the FDA’s Human Foods Program lost eighty-nine of its employees as part of the federal reduction in force, further reducing the agency’s capabilities.^354^ An influx of new safety assessment petitions, therefore, would leave the agency’s food arm overburdened.

Increased congressional appropriations are not a viable solution. As the United States Supreme Court recently noted, the congressional appropriations process forces agencies “to regularly implore Congress to fund their operations for the next year.”^355^ The process’s unpredictability prevents the agency from charting a long-term course towards increased workload and more meaningful safety review. Beyond that, although the Make America Healthy Again movement has tremendous political backing and the latest iteration of the federal government’s budget does not contemplate any cuts to FDA’s food budget (in fact, it includes some *increases* to advance MAHA’s agenda),^356^ the level of funding that the FDA would need to fill in the gaps is incompatible with the overall goal of decreasing the federal budget deficit.

User fees for all applications requiring food safety review would solve this issue. They would provide the FDA with much needed resources not only to handle any increased workload from the reforms proposed in this article, but also to meet “statutory and regulatory deadlines in its premarket review of petitions and notifications and in creating a more efficient and effective regulatory process for its post market review of substances in the food supply.”^357^ As the FDA itself has recognized, in the pharmaceutical context, user fees permit the “timely availability of innovative FDA-regulated products without compromising the agency’s commitment to scientific integrity, public health, regulatory standards, patient safety, and transparency.”^358^ Even industry groups, which originally opposed user fees for generic drugs, ultimately came to appreciate the predictability, uniformity, and general improved functioning of the process after the fees.^359^ Congress can institute fees for all food safety assessments through an act, as it has done in the past — through the Prescription Drug User Fee Act of 1992, the Generic Drug User Fee Amendment in 2012, or the Food Safety Modernization Act, which authorized the FDA to collect fees for imports, facility inspections, and third-party accreditations.^360^

User fees for food safety review could theoretically lead the FDA to become more beholden to the food industry,^361^ but ultimately such concern is misplaced for three reasons. First, paying authorization application fees is a landmark feature of our administrative system — we pay fees for zoning permits, professional licenses, and citizenship applications, to name just a few.^362^ In none of those contexts is the deciding body beholden to the applicant or more likely to grant the request because of the fee.^363^ Second, given that the FDA’s modus operandi for decades has been to seek voluntary actions by food manufacturers rather than to engage in enforcement,^364^ it is difficult to see how user fees can make the agency any less engaged than it is at present. Finally, the current system of GRAS self-determinations by paid third party scientific bodies is already ridden with *express* conflicts of interest.^365^ Any concern about user fees indirectly clouding the agency’s judgment pales by comparison.

#### 4. Require Clinical Studies

As demonstrated by both medical studies and clinical experience, food additives could have negative long-term impacts on human health through low-dose chronic exposure that does not always show up in animal studies.^366^ To ensure food safety, Congress should *require* clinical studies for any new food or color additives seeking approval through a petition process at a concern level II or above. Under the current regime, where clinical studies are never required, food manufacturers are incentivized not to track such data “due to concern about future litigation.”^367^ Indeed, many food manufacturers do not even conduct basic toxicity testing.^368^ Mandatory clinical evaluations would resolve this concern. The required studies do not need to mirror the lengthy and involved process of clinical pharmaceutical studies, because in this context there is no need to assess medical efficacy.^369^ Rather, the studies should focus only on safety, and the FDA should be empowered to promulgate a rule requiring studies that assess the most common adverse health effects from food and color additives: carcinogenicity, neurocognitive effects in children, developmental and reproductive toxicity, endocrine disruptions, and microbiome impact.^370^ The FDA should also require at least one long-term chronic toxicity study of cumulative effect of the proposed food substance, administered as a cocktail with other food additives typically found in the American diet.^371^

One of the FDA’s most important functions in assessing the safety of pharmaceuticals is precisely this role of “information production” — something the FDA can accomplish only if it has the statutory authority to *require* this type of data, rather than recommend it and hope for the best.^372^ To be sure, required clinical studies for food additives would lengthen the period of safety review for individual manufacturers, would entail a more significant investment in safety assessment, and would delay introduction of new ingredients and products on the market.^373^ Unlike a delay in life-saving vaccines or other medication, however, a delay in introducing a novel flavor of chips or cookies is not a significant burden on society and the benefit of having comprehensive safety information vastly outweighs the minor inconvenience of delayed marketing campaigns.^374^

#### 5. Require Post-Market Reporting

Finally, Congress should empower the FDA to conduct a more meaningful post-market review of food additive use and safety. In addition to the current manufacturer obligations to report acute serious adverse health effects,^375^ Congress should allow the FDA to implement an annual reporting obligation, like the one required for pharmaceutical companies.^376^ In this annual submission, food manufacturers should be required to list: (1) any new internal safety studies conducted by the manufacturer; (2) any adverse safety findings by external peer-reviewed studies; (3) any reported adverse reactions or effects (even if they do not fall within the statutory definition of “reportable foods” requiring immediate notification); and (4) total annual additive usage. These obligations would shift the onus of tracking ongoing safety findings and conducting necessary follow-up studies to the manufacturer. They would also allow the FDA to track annual food additive usage, which would in turn help it prioritize review and re-review for chemicals with higher occurrence in the typical diet.^377^

Additionally, Congress should authorize the FDA to create a reporting database for any third-party adverse testing or lab results, and an adverse report should trigger mandatory safety review and an automatic manufacturer obligation to submit additional safety information or conduct follow-up investigation, as appropriate for the type of reported event. Currently, the agency operates three adverse event reporting databases — one for pharmaceuticals (FEARS), another for dietary supplements (Safety Reporting Portal), and one for acute serious adverse events for food manufacturers.^378^ The food registry, however, is deficient in several respects. First, it is open only to manufacturers rather than to consumers or researchers.^379^ Second, it is intended only for acute serious health events,^380^ like pathogen outbreaks, but is otherwise irrelevant to food additive safety or environmental contamination. Instead of (or in addition to) this registry, Congress should authorize the creation of a database through which any third party can report an adverse health determination made by another health agency or body, such as the European Union or the World Health Organization, a peer-review study, or certified lab results showing that a food product is unsafe for human consumption. Such findings could be due to food additive toxicity, high levels of heavy metals, pathogens, environmental contaminants, production impurities, or another concern. Many such studies and tests are conducted regularly by scientific bodies, consumer protection groups, and even concerned citizens.^381^ At present, however, unless these results become the basis of prolonged litigation or a lengthy and futile petition process,^382^ they will end up in the black hole of the internet.

Ironically, the FDA bemoans its limited resources to conduct long-term safety studies or to test food products on the market.^383^ Allowing such external reporting would alleviate some of the burden currently placed on the small force of FDA researchers and field inspectors and would ensure greater transparency.

### B. Agency Actions

In lieu of (or in addition to) congressional reform, the FDA can independently make several improvements through its existing rule-making authority.

First, even without congressional abrogation of the GRAS exception, the FDA can resolve the central issues with self-certification. Indeed, on March 10, 2025, Secretary Kennedy directed Commissioner Makary to explore potential rulemaking to revise GRAS.^384^ The FDA should take this opportunity to convert the notice system from voluntary to mandatory.^385^ It should also require that manufacturers file *all* available data with their notices. Currently, “[t]he food industry does massive amounts of research that we have no access to.”^386^ That outcome should be unacceptable, and both the agency and the public should have ready access to all studies conducted on a particular substance. Lastly, the FDA should issue guidance to industry that novel substances cannot be subject to GRAS determination.^387^

Because the FDA does not have a statutorily imposed deadline for reviewing filed GRAS notices,^388^ these changes would not impact the agency’s workload significantly but would offer vastly increased transparency. To help with the eventual work of reviewing these submissions, the FDA could consider engaging artificial intelligence software to pre-assess substances and pre-categorize them in tiers of concern — such as those that pose “risks to children’s health, endocrine disruption, biomonitoring data” or have been identified as high priority by “authoritative bodies like U.S. Environmental Protection Agency (EPA), International Agency for Research on Cancer (IARC) and the National Toxicology (NTP) Program.”^389^ The same type of software could help by mining scientific databases for all studies conducted on a given substance.^390^

Second, the FDA should enforce in practice the manufacturers’ legal burden of proving safety. For both additives approved through petition and GRAS substances,^391^ the statute and regulations require the *manufacturer* to prove consensus or reasonable certainty of safety, *not* the FDA to prove *lack* of safety.^392^ In practice, however, the agency often conducts both its initial and any post-market review as if *it* has the burden to show that a substance is harmful to human health.^393^ The FDA revoked Red Dye 3’s authorization, for example, only after it affirmatively found that it “can induce cancer in male rats, through a rat specific hormonal mechanism,”^394^ even though studies casting doubt on the additive’s safety have existed for over thirty-five years. This *de facto* burden of proof that the FDA has taken upon itself taxes the agency’s resources and makes it unable to fulfill its “pre- and post-market” statutory obligations.^395^ To fix this, the agency should reverse course, demanding that manufacturers provide robust safety data with their filings or risk an automatic denial of their petition or GRAS certification. The FDA should examine all filed GRAS notices and approved food and color additives and should summarily revoke those that are filed without sufficient toxicology, feeding toxicity, and other relevant data.^396^ Likewise, the FDA should use Artificial Intelligence to identify any approved additives or noticed GRAS substances for which there are reputable, peer-reviewed studies with adverse safety results, including “findings from other authoritative institutions such as the National Institutes of Health (NIH) and the European Food Safety Authority (EFSA).”^397^ The very existence of such studies should sufficiently demonstrate that the manufacturer failed to demonstrate reasonable certainty of safety.

For future petitions and GRAS notices, the FDA should issue guidance to industry strongly recommending that manufacturers conduct long-term tests on the carcinogenic, neurocognitive, endocrine, and microbiome effect of their proposed substances to provide a more robust safety profile. The agency should also make cumulative exposure to a cocktail of food additives an explicit factor in its assessment,^398^ as it does for new drug applications.^399^ To decrease its burden of reviewing the data from these studies, the FDA should institute a graduated approach by assigning specific point values, with the highest value assigned to clinical studies in humans conducted by authoritative scientific bodies, and the lowest assigned to small-scale, short-duration lab tests funded by the manufacturer. The agency should also be more open to partnering with external reviewers; the recently announced collaboration between the FDA and NIH on the causes of “the diet-related chronic disease crisis” is a welcome start.^400^ The FDA should continue collaborating with additional research agencies to obtain similarly “‘critical information’ to inform policy,”^401^ while creating a body of external peer reviewers who could help analyze incoming safety assessments.^402^ This would simultaneously ease the agency’s workload and ensure a broader view of available scientific approaches is represented in its safety determinations.

The FDA should also promulgate rules allowing it to consider chemicals in functional groups. While the statute already mandates consideration of the cumulative dietary effect of chemically related substances in assessing safety,^403^ the agency should begin considering chemically related substances for purposes of making initial batch safety determinations and streamlining its review. In other words, if a given substance (say, PFOA) has been shown to have detrimental health effects, the FDA should consider *all* chemically related substances with common mechanism of toxicity (in this example, all long-chain PFAS) to be presumptively unsafe. The burden should then switch to manufacturers of individual substances to demonstrate that their sponsored substance behaves differently than the functional group. This burden-shifting would avoid the current whack-a-mole approach to functionally equivalent chemicals, where the FDA takes years to assess safety of an individual substance only to have the manufacturer alter a single atom and restart the clock — all while consumers continue to get sicker.

Third, the FDA should expand its mandate for lot testing to require that manufacturers conduct purity testing for environmental contaminants.^404^ Under existing regulations, environmental contaminants in food products *are* considered unsafe for human consumption, with food products considered “adulterated” if contaminants occur in excess of an action level — a limit set by the FDA, “at or above which FDA will take legal action to remove products from the market.”^405^ However, the FDA’s limited resources guarantee that most products never undergo testing unless a series of health events triggers a recall.^406^ While the FDA is currently considering partnering with states to conduct more of these safety inspections,^407^ a more efficient way to ensure that products are free from heavy metals, environmental toxins, and pathogens would be *requiring* manufacturers to test every lot, as is required for pharmaceuticals.^408^

Fourth, the FDA should mandate stricter labeling requirements.^409^ Several state bills can provide helpful insights. Texas’s House recently passed a food label bill requiring food products with ingredients banned in other countries to contain a warning, akin to the warnings for pharmaceuticals: “WARNING: This product contains an ingredient that is not recommended for human consumption by the appropriate authority in Australia, Canada, the European Union, or the United Kingdom.”^410^ A bill in New York, in a similar vein, would mandate that manufacturers disclose all GRAS ingredients on their label.^411^ The FDA should likewise require that all food and color additives be expressly listed on the label and that the manufacturer’s labeling information (on their website or otherwise available) contain data on the relevant safety testing that supports the determination that the ingredient is safe for use.^412^ This would allow not only increased agency, public, and judicial oversight, but would also help vulnerable consumers with various medical conditions, chronic medications, or other possible causes of adverse interactions to tailor their diet as medically appropriate.^413^

## VI. Conclusion

Food is both a hero and a villain in our public health story. Food is powerful medicine; it has been sustaining, nourishing, and healing people for generations. And yet, food is also the main culprit for our epidemic of diet-related diseases, cancers, childhood neurological deficits, staggering autoimmune conditions, unprecedented levels of hormonal disruptions, and shattered microbiomes. Consumers do have autonomy and bear personal responsibility to make healthier dietary choices. But the current lack of transparency and oversight has made that effectively impossible for the average consumer. With the advent of processed and ultra-processed foods, novel, lab-created, and genetically engineered commodities, dietary supplements, wellness and specialty foods, and products marketed specifically to infants and children, information asymmetry — and therefore the power dynamic — between consumers and food manufacturers has drastically shifted. To many consumers, the ingredients on product labels sound like a foreign language, making it impossible to fully appreciate the health consequences of consuming these foods. Our regulators and food manufacturers thus bear a greater responsibility to ensure that food marketed to consumers is at baseline safe. Both have increasingly failed in that task over the last century. Deceptive marketing strategies, superficial safety assessments, powerful industry lobby, and an ask-for-forgiveness-rather-than-permission mentality on the part of the manufacturers have impeded both consumers’ ability to make intentional food choices and the market’s ability to effect positive change.

The tide is turning, and food safety regulation is having its moment on the national stage. We know now more than ever that food, with its chemical complexities and capacity to cause chronic cumulative exposure, plays a vital role in public health. Legislative and regulatory reform, therefore, must increase the baseline requirements for food manufacturers, create transparency for consumers, and allow regulatory agencies to exercise robust safety review and control. It is time to ensure that our food is medicine, not poison, and that burden must rest on those who profit from food, not those who need it to live.

## Footnotes

1 Sara Moniuszko, *U.S. Spends the Most but Ranks Last in Health Compared with Other High-Income Nations, New Report Says*, CBS News (Sep. 19, 2024), https://www.cbsnews.com/news/us-ranks-last-health-despite-spending-most/ [https://perma.cc/K8V7-FUYH].

2
*See, e.g.*, *Social Determinants of Health*, U.S. Ctr. for Disease Control & Prevention (May 15, 2024), https://www.cdc.gov/public-health-gateway/php/about/social-determinants-of-health.html [https://perma.cc/LJK9-AN8Z].

3
*See, e.g.*, Varundeep Rakhra et al., *Obesity and the Western Diet: How We Got Here*, 117 Mo. Med. 536, 536-38 (2020) (describing western diet as major contributor to obesity and chronic illnesses).

4
*See* Amy-Lee Goodman, Note, *A “Natural” Stand Off Between the Food and Drug Administration and the Courts: The Rise in Food-Labeling Litigation & the Need for Regulatory Reform*, 60 B.C. L. Rev. 271, 271-72 (2019) (“Six out of ten adults in the United States suffer from a chronic disease that is linked to lifestyle and food consumption . . . .”); Sareen S. Gropper, *The Role of Nutrition in Chronic Disease*, 15 Nutrients, Jan. 2023, at 1, 1 (“Diet, often considered as a lifestyle factor, contributes to the development of many chronic conditions including obesity, cardiovascular disease, hypertension, stroke, type 2 diabetes, metabolic syndrome, some cancers, and perhaps some neurological diseases.”).

5
*National Diabetes Statistics Report*, U.S. Ctr. for Disease Control & Prevention (May 15, 2024), https://www.cdc.gov/diabetes/php/data-research/index.html [https://perma.cc/Y5U7-8BQC].

6
*See* Fang Fang Zhang et al., *Preventable Cancer Burden Associated with Poor Diet in the United States*, 3 JNCI Cancer Spectrum 1, 1-7 (2019).

7 U.S. Ctr. for Disease Control & Prevention, Chronic Kidney Disease in the United States, 2023, at 1 (2023), https://www.cdc.gov/kidney-disease/media/pdfs/CKD-Factsheet-H.pdf [https://perma.cc/2F2T-BF4T].

8
*See, e.g.*, *Unhealthy Diets and Malnutrition*, NCD Alliance, https://ncdalliance.org/why-ncds/risk-factors-prevention/unhealthy-diets-and-malnutrition [https://perma.cc/GVK5-EVAS] (last visited Sep. 6, 2025) (describing how unhealthy diets interact with noncommunicable diseases).

9
*See, e.g.*, *Food and Chemical Exposure*, Mount Sinai Inst. for Exposomic Rsch. (Feb. 20, 2025), https://mountsinaiexposomics.org/food-and-chemical-exposure/ [https://perma.cc/YWQ9-FAQ4] (describing food as a major source of nutrition but also chemical exposure).

10
*See, e.g.*, Claire McCarthy, *Common Food Additives and Chemicals Harmful to Children*, Harv. Health Publ’g (July 26, 2021), https://www.health.harvard.edu/blog/common-food-additives-and-chemicals-harmful-to-children-2018072414326 [https://perma.cc/6DM2-DMSP] (describing the various chemicals in food and potential harms each one may cause especially to children).

11
*See* Michael Merschel, *Too Much of a Food Thing: A Century of Change in How We Eat*, Am. Heart Ass’n (Mar. 13, 2024), https://www.heart.org/en/news/2024/03/13/too-much-of-a-food-thing-a-century-of-change-in-how-we-eat [https://perma.cc/7VHA-LN2C] (describing how industrialization has changed the food industry, how Americans eat, and their health).

12
*See, e.g.*, Bruce Bower, *A Surprising Food May Have Been a Staple of the Real Paleo Diet: Rotten Meat*, Sci. News (Mar. 29, 2023), https://www.sciencenews.org/article/meat-rotten-putrid-paleo-diet-fire-neanderthal [https://perma.cc/9C7U-FVEA] (describing evidence dating back to early humans often eating putrid foods that contained numerous microorganisms); Guadalupe Monge et al., *Earliest Evidence of Pollution by Heavy Metals in Archaeological Sites*, 5 Sci. Rep., Sep. 21, 2015, at 1, 1-5 (describing evidence taken from cave soil showing high heavy metals exposure in early human archeological sites).

13
*See* Joe Schwarcz, *The Beginnings of Chemical Synthesis*, McGill (Oct. 22, 2024), https://www.mcgill.ca/oss/article/technology-history-did-you-know/beginnings-chemical-synthesis [https://perma.cc/3VKV-6J7C] (describing the history of chemical synthesis and how it came to be from the early 19^th^ century).

14
*See* Lars Noah & Richard Merrill, *Starting from Scratch?: Reinventing the Food Additive Approval Process*, 78 B.U. L. Rev. 329, 332 (1998).

15
*See generally* Merschel, *supra* note 11 (discussing the advent of frozen and processed foods and the conveniences and dangers that came with them).

16 Ari Shapiro, *How A 19th Century Chemist Took on The Food Industry with A Grisly Experiment*, NPR (Oct. 8, 2018), https://www.npr.org/sections/thesalt/2018/10/08/654066794/how-a-19th-century-chemist-took-on-the-food-industry-with-a-grisly-experiment [https://perma.cc/5D8F-DCJT].

17
*See* Pure Food and Drugs Act, Pub. L. No. 59-384, 34 Stat. 768 (1906) (allowing for greater regulation of “adultered … foods, drugs, [and] medicines”).

18 Food, Drug, and Cosmetics Act, Pub. L. No. 75-717, 52 Stat. 1040 (1938).

19 Noah & Merrill, *supra* note 14, at 335-36.

20
*Id.*

21 Food Additives Amendment, Pub. L. No. 85-929, 72 Stat. 1784 (1958).

22 21 U.S.C. § 348.

23
*See infra* Part III.

24
*See, e.g.*, Helena Bottemiller Evich, *The FDA’s Food Failure*, Politico (Apr. 8, 2022), https://www.politico.com/interactives/2022/fda-fails-regulate-food-health-safety-hazards/ [https://perma.cc/BZ45-WW6J] (describing the inefficiency of the FDA, lack of staffing, clear priorities, and industry lobbying affecting the agency).

25
*See, e.g.*, Noah & Merrill, *supra* note 14, at 330; Laurie J. Beyranevand, *Generally Recognized as Safe?: Analyzing Flaws in the FDA’s Approach to GRAS Additives*, 37 Vt. L. Rev. 887, 888-89, 921-22 (2013) (analyzing how the FDA has lagged in regulating food by not providing clear guidance, limits on potentially harmful additives, or defining “harmful”).

26 Donald E. Metz, *Food, Nutrition, and Health.* 44 Health Affs. 381, 381 (2025) (“Growth in diet-related chronic disease and its consequences has spurred increased interest in diet and lifestyle interventions to improve access to healthy, safe, and affordable food; eradicate hunger; and reduce health disparities.”).

27
*See, e.g.*, Linley Sanders, *What US Adults Think of Robert F. Kennedy Jr. and His Views on Vaccines, Fluoride and Raw Milk*, The Associated Press (Jan. 29, 2025), https://apnews.com/article/rfk-jr-poll-fluoride-vaccinations-milk-abortion-88744534e2371bcfd0cd0b67fd659bd0 [https://perma.cc/X7NY-RXW9] (“About two-thirds of Americans “somewhat” or “strongly” favor restricting or reformulating processed foods to remove ingredients like added sugar or dyes. This is an area where Democrats and Republicans agree.”); Taylor Orth, *What Americans Think of RFK Jr.’s Make America Healthy Again Agenda*, YouGov (Dec. 13, 2024), https://today.yougov.com/politics/articles/51140-what-americans-think-of-rfk-jrs-make-america-healthy-again-agenda [https://perma.cc/BZJ7-XUXW]; David Hilzenrath, *How the FDA Opens the Door to Risky Chemicals in America’s Food Supply FDA’s Limited Oversight on Food Additives Raises Health Concerns*, KFF Health News (Mar. 10, 2025), https://kffhealthnews.org/news/article/food-ingredients-chemicals-additives-fda-oversight-lax-industry-honor-system/ [https://perma.cc/V7SJ-W9HK].

28
*See, e.g.*, Metz, *supra* note 26, at 381. *See also NASDA Members Advocate for Expansion of “Food As Medicine” Programs*, Nat’l Ass’n of State Dep’ts of Agric. 1 (Feb. 26, 2025), https://www.nasda.org/nasda-members-advocate-for-expansion-of-food-as-medicine-programs/?trk=feed_main-feed-card_feed-article-content [https://perma.cc/R52U-9KVS] (noting that the National Association of State Departments of Agriculture voted in favor of “food as medicine” initiatives); *Rockefeller Foundation Invests $3.5 Million to Support American Farmers, Improve Nutrition & Address Chronic Disease*, The Rockefeller Found. 1-2 (Feb. 13, 2025), https://www.rockefellerfoundation.org/news/rockefeller-foundation-invests-3-5-million-to-support-american-farmers-improve-nutrition-address-chronic-disease/?trk=feed_main-feed-card_feed-article-content [https://perma.cc/8C99-C4YK] (referencing both the donation from the Rockefeller Foundation and the “growing market” of Food Is Medicine in the United States).

29
*See, e.g.*, Helena Bottemiller Evich, ‘*Make America Healthy Again’ Is Officially in the Cabinet — What Comes Next?*, FoodFix (Feb. 14, 2025), https://foodfix.co/make-america-healthy-again-secures-a-place-in-the-cabinet-what-comes-next/ [https://perma.cc/SK8P-FKHL]. *See also* Hilzenrath, *supra* note 27 (“At his confirmation hearing, Makary said some ingredients cause a chronic, low-grade inflammatory reaction in the gastrointestinal tract. ‘And what are we doing? We are drugging our nation’s children at scale,’ he said.”).

30 Alexander Tin, *FDA Stops Short of Synthetic Food Dye Ban, Calls on Industry to Stop Use*, CBS News (Apr. 23, 2025, at 07:51 ET), https://www.cbsnews.com/news/fda-artificial-food-dyes-rfk-jr/?trk=feed_main-feed-card_ingested-content-summary-external-video-content [https://perma.cc/X34L-DSH5].

31
*See, e.g.*, Will Kubzansky & Rachel Cohrs Zhang, *Food Industry Says There’s No Agreement with US Health Agency to Cut Dyes*, Bloomberg (Apr. 23, 2025, at 14:55 ET), https://www.bloomberg.com/news/articles/2025-04-23/food-industry-says-there-s-no-agreement-with-hhs-to-cut-dyes?trk=feed_main-feed-card_feed-article-content&embedded-checkout=true [https://perma.cc/BXC8-GEUE]. *See also* Meghan Enslow, *Food Companies Fail on Clean Label Promises*, Ctr. for Sci. in the Pub. Int. (Jan. 2, 2025), https://www.cspinet.org/cspi-news/food-companies-fail-clean-label-promises?trk=feed_main-feed-card_feed-article-content [https://perma.cc/U5UR-JEP4] (noting that food companies have promised cutting food additives and dyes before and have failed to deliver on these voluntary commitments).

32
*See generally* Off. Disease Prevention & Health Promotion, U.S. Dep’t of Health & Hum. Servs., Food Is Medicine Landscape Summary 2-3 (2024), https://odphp.health.gov/sites/default/files/2024-09/Food%20Is%20Medicine%20Landscape%20Summary%20FINAL%20508.pdf [https://perma.cc/Y7DH-JZCB] (describing the general definition and important principles of Food Is Medicine).

33 Ronit Ridberg et al., *‘Food Is Medicine’ in the US: A National Survey of Public Perceptions of Care, Practices, and Policies*, 44 Health Affs. 398, 398-400 (2025).

34
*See generally* Lejla Zunic, Armin Skrbo & Amra Dobraca, *Historical Contribution of Pharmaceutics to Botany and Pharmacognosy Development*, 29 Mater Sociomed. 291, 291 (2017) (providing a historical account of the development of pharmaceuticals from plants); *History of Medicine*, Britannica (Apr. 25, 2025), https://www.britannica.com/science/history-of-medicine [https://perma.cc/X3ZV-PYEX] (describing the use of plants, animal products, and minerals as forms of medicine throughout history as well as revolutionary discoveries in the 19th century, like anesthesia, that helped redefine medical procedures).

35 Laurence Totelin, *When Foods Become Remedies in Ancient Greece: The Curious Case of Garlic and Other Substances*, 160 J. Ethnopharmacol. 30, 30 (2015).

36
*See* Stephen Carpenter et al., An Interlinear Transliteration and English Translation of Portions of The Ebers Papyrus Possibly Having to Do with Diabetes Mellitus 6 (2006); *The Nutriment of Hippocrates*, Quintus Curtius (Feb. 13, 2021), https://qcurtius.com/2021/02/13/the-nutriment-of-hippocrates/ [https://perma.cc/J8TW-KHL9].

37
*See, e.g.*, Unnikrishnan Payyappallimana & Padma Venkatasubramanian, *Exploring Ayurvedic Knowledge on Food and Health for Providing Innovative Solutions to Contemporary Healthcare*, Frontiers Pub. Health, Mar. 31, 2016, at 1, 1-2; Yuan Haidan et al., *The Traditional Medicine and Modern Medicine from Natural Products*, Molecules, Apr. 29, 2016, at 1, 1-2.

38
*See, e.g.*, Rich Guylas, *Revolutionary War and 19th Century Plants and Herbal Remedies*, Penn State Extension (Mar. 20, 2022), https://extension.psu.edu/programs/master-gardener/counties/bradford/news/revolutionary-war-and-19th-century-plants-and-herbal-remedies#:~:text=County%20%E2%80%94%20Master%20Gardener-,Revolutionary%20War%20and%2019th%20Century%20Plants%20and%20Herbal%20Remedies,other%20ailments%20common%20even%20today [https://perma.cc/4RK6-NKKY] (discussing the use of herbal medicines in North America during the Revolutionary War, with the knowledge of those remedies originating from Native Americans and immigrants from other countries). *See generally* Juliana Adelman, *Invalid Cookery, Nursing and Domestic Medicine in Ireland, c. 1900*, 73 J. Hist. Med. Allied Sci. 188, 191-93 (2018) (describing the tradition of domestic medicine in Ireland, which was heavily reliant on gastronomy and food).

39
*See, e.g.*, Hit Kishore Goswami & Hitendra Kumar Ram, *Ancient Food Habits Dictate that Food Can Be Medicine but Medicine Cannot Be “Food”!!*, Meds., Nov. 13, 2017, at 1, 1-2 (discussing both the process of ancient people for understanding which plants are safe or poisonous for consumption, and the fact that medicinal substances in plants, when overconsumed, could lead to “chronic diseases including aging, cancer, cardiovascular diseases, rheumatoid arthritis, atherosclerosis, etc.”); Frank Erbguth, *Historical Notes on Botulism*, *Clostridium Botulinum, Botulinum Toxin, and the Idea of the Therapeutic Use of the Toxin*, 19 Mov. Disords. S2, S3-S4 (2004) (discussing historical notes about the process of investigating instances of food poisoning and various food pathogens, including botulism).

40
*See, e.g.*, sources cited *supra* note 39.

41
*Food Is Medicine: A Project to Unify and Advance Collective Action*, Off. of Disease Prevention & Health Promotion (July 14, 2025), https://odphp.health.gov/our-work/nutrition-physical-activity/food-medicine [https://perma.cc/P46R-X7ZF].

42
*Id.*

43
*Id.*

44
*Id.*

45
*See Types of Interventions*, Off. of Disease Prevention & Health Promotion (Sep. 12, 2024), https://odphp.health.gov/foodismedicine/understanding-food-medicine/types-interventions [https://perma.cc/J76M-FRDD] (referencing different federal Food Is Medicine interventions, such as federal nutrition assistance and how people may also access Food Is Medicine interventions through state-based programs).

46 Ridberg et al., *supra* note 33, at 398

47
*See* Erika Hanson et al., *The Evolution and Scope of Medicaid Section 1115 Demonstrations to Address Nutrition: A US Survey*, Health Affs. Jan. 30, 2024, at 1, 1 (referencing that states are using Medicaid 1115 demonstration waivers to provide coverage for Food Is Medicine Initiatives).

48 Ridberg et al., *supra* note 33, at 398.

49
*See id.* (referencing how poor nutrition and diet can cause diseases); *Healthy Diet*, World Health Org. (Apr. 29, 2020), https://www.who.int/news-room/fact-sheets/detail/healthy-diet [https://perma.cc/X83K-AUN2] (noting that the excessive consumption of fats, sugars, and sodium is unhealthy and that fruits and vegetables are a part of a healthy diet); Jenette Restivo, *High-protein Foods: The Best Protein Sources to Include in a Healthy Diet*, Harv. Health Publ’g (Dec. 1, 2023), https://www.health.harvard.edu/nutrition/high-protein-foods-the-best-protein-sources-to-include-in-a-healthy-diet [https://perma.cc/UZS7-M8WT] (explaining that protein is a part of a healthy diet).

50
*See generally* Katya S. Cronin, *A One Health Approach to Healthy Food*, 91 Brook. L. Rev. (forthcoming Dec. 2025) (noting that the federal government defines what constitutes “healthy food” too narrowly and that even “healthy food” can be dangerous because of problems like intensive farming practices) [hereinafter Cronin, *One Health*]. *See also* Reagan-Udall Found. for the FDA, Real-world Data to Assess Long-term Impact of FDA Food-Related Regulations and Policies 4 (2024), https://reaganudall.org/sites/default/files/2024-10/100324_Food%20Evidence%20Generator_FINALpdf.pdf [https://perma.cc/6EJM-FBJ9] (“Food that includes hazards like pathogens, heavy metals, foreign materials, or undeclared allergens cannot provide good nutrition.”).

51
*See* Jonel Aleccia, *Americans Get More Than Half Their Calories from Ultra-Processed Foods, CDC Report Says*, The Associated Press (Aug. 7, 2025) (noting that despite heightened awareness of the danger or processed foods, adults consume nearly half of their calories through these foods).

52
*See* Alan Wayne Jones, *Early Drug Discovery and the Rise of Pharmaceutical Chemistry*, 3 Drug Testing Analysis 337, 337 (2011) (noting that the first pharmaceutical drug was discovered in 1869).

53
*Id.*

54
*See* T.R. Bradshaw, *The Use of Salicylic Acid as a Food Preservative*, 161 Lancet 717, 717-18 (1903).

55 U.S. Food & Drug Admin., Chemicals in Food: The Facts 1 (2024); Muhammad Tuhin, *How Medicines Work in the Body: A Scientific Overview*, Sci. News Today (May 14, 2025), https://www.sciencenewstoday.org/how-medicines-work-in-the-body-a-scientific-overview [https://perma.cc/W9G7-LUN9]; Connie M. Weaver et al., *Processed Foods: Contributions to Nutrition*, 99 Am. J. Clin. Nutr. 1525, 1529 (2014).

56
*Botulism*, World Health Org. (Sep. 25, 2023), https://www.who.int/news-room/fact-sheets/detail/botulism/ [https://perma.cc/K5XW-V7VL] (noting that poorly preserved foods are a source of botulism, which is a type of bacteria, and that botulism is rare); *Preservatives*, ChemicalSafetyFacts (Oct. 14, 2022), https://www.chemicalsafetyfacts.org/chemicals/preservatives/ [https://perma.cc/U4AW-EP6L] (highlighting that preservatives are used to help prevent food spoilage caused by bacteria); *Scurvy*, Cleveland Clinic (Oct. 20, 2022), https://my.clevelandclinic.org/health/diseases/24318-scurvy [https://perma.cc/JSV4-AR8D] (explaining that scurvy is caused by a vitamin C (ascorbic acid) deficiency and that severe scurvy is rare); Ocean Robbins, *Common Food Additives in Natural & Organic Foods: Are They Safe?*, Food Revolution Network (May 14, 2021), https://foodrevolution.org/blog/common-food-additives-natural-organic/ [https://perma.cc/8Y4K-MNK7] (noting that ascorbic acid is one of the most common food additives); *Neural Tube Defects (NTD)*, Cleveland Clinic (Mar. 30, 2022), https://my.clevelandclinic.org/health/diseases/22656-neural-tube-defects-ntd [https://perma.cc/WQ7B-XF8F] (referencing that neural tube defects are linked to a folic acid deficiency and occur in about 3,000 pregnancies each year in the United States); *The Ups and Downs of Folic Acid Fortification*, Harv. Health Publ’g (Mar. 1, 2008), https://www.health.harvard.edu/womens-health/the-ups-and-downs-of-folic-acid-fortification [https://perma.cc/6F9V-2PHE] (explaining that folic acid is synthetic and is added to grains and cereals).

57
*See* Bradshaw, *supra* note 54 (investigating whether ingesting salicylic acid can be harmful to human health).

58
*See* John P. Swann, *FDA’s Origin*, U.S. Food & Drug Admin. (June 23, 2014), https://www.fda.gov/about-fda/changes-science-law-and-regulatory-authorities/fdas-origin [https://perma.cc/AY46-864E].

59 Carrie A. Scrufari, *Substances Generally Recognized as Safe - Until They’re Not: Challenges in Protecting the Food Supply in a Processed World*, 36 Stan. Env’t. L.J. 219, 224 (2017).

60 21 U.S.C. § 348(c)(5)(B).

61 Iris Myers, *EWG’s Dirty Dozen Guide to Food Chemicals: The Top 12 to Avoid*, Env’t Working Grp. (Mar. 18, 2025), https://www.ewg.org/consumer-guides/ewgs-dirty-dozen-guide-food-chemicals-top-12-avoid [https://perma.cc/J5JN-P3W5].

62
*See, e.g.*, *Ultra-Processed Foods Are Sabotaging Your Diet. Here’s How to Cut Them Out*, Henry Ford Health (Sep. 29, 2023), https://www.henryford.com/blog/2023/09/ultra-processed-foods [https://perma.cc/MA8R-A87F].

63
*Id.*

64 Myers, *supra* note 61.

65
*Id.*

66
*See id.*

67 Samira Choudhury et al., *Research on Health Impacts of Chemical Contaminants in Food*, 100 Bull. World Health Organ. 180, 180 (Mar. 1, 2022) (listing chemical contaminants that exist in food today).

68 Francis S. Collins, *Exceptional Opportunities in Medical Science: A View from the National Institutes of Health*, 313 [J]AMA 131, 131 (2015) (Noting that “[s]cientific and technological breakthroughs that have arisen from NIH-supported research account for many of the gains that the United States has seen in health and longevity.”).

69
*See* Omar Israel González Peña et al., *Pharmaceuticals Market, Consumption Trends and Disease Incidence Are Not Driving the Pharmaceutical Research on Water and Wastewater*, 18 Int. J. Env’t. Res. Pub. Health, Mar. 4, 2021, at 1, 2 (noting that the largest markets by percentage of drugs consumed include musculoskeletal drugs, cardiovascular, oncological, and anti-infective drugs, and drugs for chronic metabolic disorders ranks fifth, despite being the fastest growing).

70
*See, e.g.*, Craig I. Coleman et al., *Dosing Frequency and Medication Adherence in Chronic Disease*, 18 J. Managed Care Pharm. 527, 528 (2012) (concluding that while frequency of dosing for chronic conditions fell between once and four times daily, once-daily doses had the highest adherence).

71
*See* Regan L. Bailey et al., *Frequency of Eating in the US Population: A Narrative Review of the 2020 Dietary Guidelines Advisory Committee Report*, Nutritional Epidemiology & Pub. Health, Feb. 26, 2025, at 1, 2.

72 Hilzenrath, *supra* note 27. *See also* U.S. Food & Drug Admin., Redbook 2000: Guidance for Industry and Other Stakeholders Toxicological Principles for the Safety Assessment of Food Ingredients 179 (2007) [hereinafter Redbook 2000] (“Given the nature of consumption patterns of food ingredients (i.e., chronic, lifetime exposures), it is necessary to require the chronic safety testing of food ingredients that would be representative of lifetime exposure in humans.”).

73
*See* Amanda E. Miller & Samar Nicolas, *Federal Regulation of Medication Dispensing*, StatPearls Publ’g (2023), https://www.ncbi.nlm.nih.gov/books/NBK582130/ [https://perma.cc/4K5D-575Z].

74
*See, e.g.*, Raila Cinda Brejt, *Food Regulation and the Nondisclosure of Ingredients: Ignorance Is Not Always Bliss*, 33 Health Law. 38, 38-39 (2021) (recounting the case of a patient battling “Clostridium Difficile (c-diff.), a bacterium that causes gastrointestinal disturbances and potentially deadly inflammation of the gastrointestinal tract,” who experienced worsening symptoms because a food additive in her diet — trehalose — encouraged hypervirulence of c-diff and inhibited the effect of the vancomycin the patient was taking).

75
*See, e.g.*, Editorial, *Medication is a Necessity, Not an Option*, Daily News (Mar. 31, 2025, at 16:00 ET), https://www.dailynews.lk/2025/03/31/editorial/752646/medication-is-a-necessity-not-an-option/ [https://perma.cc/82NQ-SHF2].

76
*See* Scrufari, *supra* note 59, at 267 (arguing that the doctrine of administrative necessity should not apply to permit potentially carcinogenic food ingredients because neither color nor food additives are necessary for safe food production).

77 Frank C. Woodside & Margie Searcy-Alford, Drug Product Liability § 15A.03 (2025). *See also* U.S. Food & Drug Admin., *The FDA’s Drug Review Process: Ensuring Drugs Are Safe and Effective*, https://www.fda.gov/drugs/information-consumers-and-patients-drugs/fdas-drug-review-process-ensuring-drugs-are-safe-and-effective [https://perma.cc/TQ48-9WSC] (last visited June 21, 2025) [hereinafter *Review Process*].

78 Amy Kapczynski, *Dangerous Times: The FDA’s Role in Information Production, Past and Future*, 130 Minn. L. Rev. 2357, 2358 (2018).

79
*See, e.g.*, Rachel E. Sachs et al., *Rethinking Innovation at FDA*, 104 B.U. L. Rev. 513, 534 (2024) (comparing the FDA efforts to spur rare disease innovation with the inherent barriers set by FDA-established exclusivity periods); Daniel Aaron et al., *The FDA Struggle to Withdraw Makena: Problems With the Accelerated Approval Process*, 328 [J]AMA 2394, 2394 (2022), (detailing how the FDA’s haste to approve a new drug lead to difficulty withdrawing approval after the drug’s dangers became known); Jordan A. Marzzacco, *A Dose of Reality: The Deadly Truth About Federal Preemption of Generic Drug Manufacturer Liability*, 24 Widener L.J. 355, 360-62, 367 (2015) (cautioning that the new drug approval process allows for the subsequent approval of dangerous generic drugs); Robert G. Pinco, *Implications of FDA’s Proposal to Include Foreign Marketing Experience in the Over-the-Counter Drug Review Process*, 53 Food Drug L.J. 105, 105, 113 (1998) (warning that the NDA process is too costly and complex for “older nonpatented products” and products with multiple active constituents).

80
*See* 21 C.F.R. §§ 314.125(b)(2)-(4), 314.127(a)(8)(i) (2025) (stating that the FDA will refuse to approve NDAs and ANDAs for marketing purposes if there is inadequate safety review or evidence from such a review that a product is unsafe); Ana M. B. Amorim et al., *Advancing Drug Safety in Drug Development: Bridging Computational Predictions for Enhanced Toxicity Prediction*, 37 Chem. Rsch. Toxicol. 827, 843-44 (2024) (reiterating the continued importance of safety assessments in the modern age of drug development); Catherine T. Struve, *The FDA and the Tort System: Postmarketing Surveillance, Compensation, and the Role of Litigation*, 5 Yale J. Health Pol’y, L., & Ethics 587, 588 (2005) (emphasizing the need for FDA safety reviews to protect consumer welfare).

81 Woodside & Searcy-Alford, *supra* note 77, § 8.02.

82
*See* U.S. Food & Drug Admin., Nonclinical Safety Studies for the Conduct of Human Clinical Trials and Marketing Authorization for Pharmaceuticals 2 (2010). *See also* 21 C.F.R. § 312.23 (2025).

83 Jules T. Mitchel, *FDA Relations During Drug Development*, 2 Dialogues in Clinical Neuroscience 213, 213 (2000).

84 Kapczynski, *supra* note 78, at 2364-65.

85
*Review Process*, *supra* note 77.

86
*See, e.g.*, Militrano v. Lederle Labs., 3 Misc. 3d 523, 539 (Kings Cnty. Sup. Ct. 2003) (stating that the investigation and testing necessary to prepare and approve an investigational new drug application tends to take eighteen months); Mukesh Kumar & Janice Longstreth, *Risks and Benefits of Conducting Preclinical Studies in the Global Setting*, Reg. Focus, Dec. 2011, at 20, 21 (2011), https://fdamap.com/wp-content/uploads/2025/03/preclinical_testing.pdf [https://perma.cc/FDA6-EUHU] (“Anywhere from 10 to 50 preclinical studies may be required just to establish a given product’s safety profile.”).

87 Michael D. Green, *Statutory Compliance and Tort Liability: Examining the Strongest Case*, 30 U. Mich. J.L. Reform 461, 486 (1997).

88
*See* 21 C.F.R. § 312.32(c)(1)(i) (2025).

89 Green, *supra* note 87, at 486.

90
*See* 21 C.F.R. § 312.23 (2025).

91
*See* 21 C.F.R. § 312.23(a)(3) (2025).

92
*See* 21 C.F.R. §§ 312.21, 312.40(b) (2025); U.S. Food & Drug Admin., Institutional Review Boards Frequently Asked Questions 58 (2025).

93 21 C.F.R. § 312.21 (2025). *See also* Abigail All. for Better Access to Developmental Drugs v. Von Eschenbach, 495 F.3d 695, 698 (D.C. Cir. 2007) (stating that a Phase 1 study primarily focuses on safety, typically includes twenty to eighty subjects, seeks to determine metabolism and pharmacologic actions of the drug, and assesses side effects of increasing doses).

94
*Review Process*, *supra* note 77.

95
*Id.*

96 21 C.F.R. § 312.21(b) (2025); *see* 21 C.F.R. § 312.23 (a)(6)(iii)(e) (2025) (planned maximum dosage).

97 21 C.F.R. § 312.21(c) (2025).

98
*See Review Process*, *supra* note 77.

99
*See* 21 C.F.R. § 312.22(a) (2025).

100 495 F.3d at 698. *See also* 21 C.F.R. § 312.32(c)(1)(i)(A) (2025) (stating that an adverse reaction must be reported only if evidence suggests a causal relationship between the drug and the adverse event, such as an isolated occurrence of an uncommon event that is known to be strongly associated with drug exposure); 21 C.F.R. § 312.42(b) (2025) (listing the possible reasons for a clinical hold of a phase under an IND, including exposure to “an unreasonable and significant risk of illness or injury”).

101 Militrano, 3 Misc. 3d at 540.

102
*Review Process*, *supra* note 77.

103
*See* 21 U.S.C. § 355(a).

104 21 U.S.C. § 355(b)(1)(A)(i)-(vi).

105
*See* 21 U.S.C. §§ 355(b)(1)(A)(vii), 355(c).

106 Militrano, 3 Misc. 3d at 540. Instead of a complete NDA, sponsors may submit an Abbreviated New Drug Application (ANDA) for the approval of generic drugs that are bioequivalent to and have the same rate and extent of absorption into the bloodstream as an already approved innovator drug. *See* 21 U.S.C. § 355(j).

107
*See* 21 U.S.C. § 355(c)(1)(B).

108 Prescription Drug User Fee Rates for Fiscal Year 2025, 89 Fed. Reg. 61474, 61480 (July 31, 2024).

109
*See The GRAS Is Not Always Greener: Why GRAS Status Does Not Guarantee Excipient Safety*, Premier Consulting (June 21, 2016), https://premierconsulting.com/resources/blog/the-gras-is-not-always-greener/ [https://perma.cc/AM9M-DSSN] (“[T]he focus of sponsors is often primarily on supporting the safety and efficacy of the active ingredient(s). However, the safety of the *inactive* ingredients (excipients) needs to be supported as well.”).

110 21 C.F.R. § 314.50(d)(1)(i)-(ii) (2025).

111
*See* 21 C.F.R. § 314.50(d)(5)(vi) (2025). *See also* 21 C.F.R. § 314.3(b) (2025) (defining “drug product”).

112
*See* 21 C.F.R. § 314.50(f)(3) (2025). The only inert ingredients in drugs regulated separately are color additives. *See* 21 C.F.R. pt. 70 (2025). For color additives used in drugs, sponsors must specifically petition for the use of the additive, denoting the chemical composition of the additive, the probable level of additive consumption, the degree of exposure to the additive, and the intended level of use of the additive. *See* 21 C.F.R. § 71.1(c) (2025). The maximum percentage of color additives may not exceed 1/100th of the “maximum “no-effect level” found when testing the additive in animals. *See* 21 C.F.R. § 70.40 (2025).

113
*See* 21 U.S.C. § 355(d) (noting determination of safety and effectiveness “requires that there be evidence consisting of adequate and well controlled investigations including clinical investigations, by experts qualified by scientific training and experience”).

114
*See* Woodside & Searcy-Alford, *supra* note 77, § 8.02 (discussing that “potential harm must be balanced against the potential benefits to be realized from a given drug”); U.S. Food & Drug Admin., Benefit-Risk Assessment for New Drug and Biological Products: Guidance for Industry 4 (2023) (explaining that, in cases of challenging benefit-risk assessments, approval may be granted by identifying a subpopulation characterized by disease “for whom benefits outweigh the risk even if they do not do so in a broader population”).

115 21 C.F.R. § 201.100(d) (2025).

116
*See* Richard A. Merrill, *The Architecture of Government Regulation of Medical Products*, 82 Va. L. Rev. 1753, 1756-57, 1853-54 (1996) (discussing that current procedures required for FDA approval of new drugs, including a required review of evidence regarding the safety and effectiveness of every use claimed in labeling of new drug, is one of the many obstacles to achieving full FDA approval).

117
*See* Philip S. Goldberg et al., *A Prescription for Pharmaceutical Preemption*, 20 Rutgers J.L. & Pub. Pol’y 209, 218-19 (2023) (explaining that if during post-market surveillance, clinically significant hazard is discovered NDA-holders “must revise a drug’s labeling to include appropriate warning for that hazard”).

118 21 C.F.R. § 314.70(c)(6)(iii)(A) (2025).

119
*See* 21 U.S.C. § 353(d) (explaining that “[i]f the secretary determines, with respect to a covered drug, that the available scientific evidence meets the standards under section 355 of this title for adding or modifying information to the labeling … the Secretary may initiate the process” of updating such labels).

120 21 U.S.C. § 355(e), (k)(1).

121 21 C.F.R. § 314.81(b) (2025).

122 21 C.F.R. § 201.57(c)(6)(i) (2025).

123 21 U.S.C. §§ 355(o)(3)(C), 355-1(b)(3). *See also* Kapczynski, *supra* note 78, at 2371 (“FDA reviewers may also query applicants for more data and dig deeper into the record to validate and—if needed—correct data.”).

124 21 U.S.C. § 355-1(b)(3). *See also* 21 U.S.C. § 355(k)(4) (discussing collaborations with public, academic and private entities to conduct research and analysis of post market drug safety and to allow for prompt investigation of newly-approved drugs).

125 21 U.S.C. § 355-1(b)(3).

126 21 U.S.C. § 355-1(a)(2)(A).

127
*See, e.g.*, 21 U.S.C. § 333(f)(4) (civil sanctions); 21 U.S.C. § 333(a) (criminal misdemeanor penalties); 21 U.S.C. § 355(e) (withdrawing drug approval given new evidence of lack of safety).

128
*See* 21 C.F.R. § 211.42 (2025) (discussing requirements for buildings used in manufacture, processing, packaging, or holding of drug products); 21 C.F.R. §§ 211.46, 211.56 (2025) (describing ventilation, air filtration, and sanitation requirements falling under good manufacturing practice for finished pharmaceuticals).

129 21 C.F.R. §§ 211.160, 211.110 (2025).

130 21 C.F.R. § 211.84 (2025).

131 21 C.F.R. § 211.165 (2025).

132
*See, e.g.*, Matthew Herder, *Aducanumab, Accelerated Approvals & the Agency: Why the FDA Needs Structural Reform*, 51 J.L., Med. & Ethics 900, 902 (2024) (describing clinical trials verifying safety of Mylotarg for acute myeloid leukemia ended prior to completion years after FDA approval of drug because no clinical benefit was shown and number of trial participants died due to treatment toxicity); Daniel G. Aaron, *The Fall of FDA Review*, 22 Yale J. Health Pol’y, L., & Ethics 95, 130-31 (2023) (arguing that accelerated approval results in FDA allowing less reliable drugs to come to market by holding them to reduced standards with no easy way to withdraw such drugs should they fail confirmatory trials); Aaron et al., *supra* note 79, at 2394 (discussing delay between accelerated approval of preterm birth prevention drug Makena in 2011 to confirmatory trial results, which did not confirm clinical benefit in 2019).

133
*See* Sachs et al., *supra* note 79, at 543-47 (describing how FDA approval guidance often requires large clinical trials which increase costs and disincentivize innovation, and highlighting how lack of flexibility in approving drugs for ultra-rare diseases acts as an implication for innovation incentives); W. Nicholson Price II, *Making Do in Making Drugs: Innovation Policy and Pharmaceutical Manufacturing*, 55 B.C. L. Rev. 491, 494-95 (2014) (explaining that patents and FDA action leading to periods of market exclusivity for new drugs as well as regulatory barriers slow manufacturing innovation causing firms to avoid introducing new technologies into the market).

134
*See* Catherine M. Sharkey & Daniel J. Kenny, *FDA Leads, States Must Follow*, 102 Wash. U. L. Rev. 155, 168-71 (2024) (indicating that ruling in *Loper Bright* has not reduced deference given to FDA’s extensive scientific expertise and safety assessment regulations); *Review Process*, *supra* note 77 (describing that FDA’s evaluation process as one that is rigorous, scrutinizing every characteristic of new drugs); 21 C.F.R. §§ 314.125(b)(2)-(4), 314.127(a)(8)(i) (2025) (explaining FDA’s authority to refuse NDAs and ANDAs if information suggests drug product is unsafe).

135 Hilzenrath, *supra* note 27.

136
*See, e.g.*, Katharine Van Tassel, *The Introduction of Biotech Foods to the Tort System: Creating a New Duty to Identify*, 72 U. Cin. L. Rev. 1645, 1651 (2004) (noting that whole or traditional foods have only manufacturing and labeling requirements, but no certification or safety testing requirements).

137 Ctr. for Food Safety v. Becerra, 565 F. Supp. 3d 519, 534 (S.D.N.Y. 2021).

138 21 U.S.C. § 393(b)(2); *See FDA Update on Post-market Assessment of Chemicals in the Food Supply*, U.S. Food & Drug Admin. (Aug. 19, 2025), https://www.fda.gov/food/hfp-constituent-updates/fda-update-post-market-assessment-chemicals-food-supply-0 [https://perma.cc/4QE4-28W9] (discussing recent FDA initiatives to improve food chemical oversight including of food additives and color additives).

139 21 U.S.C. § 321(s).

140
*Types of Food Ingredients*, U.S. Food & Drug Admin. (July 6, 2023), https://www.fda.gov/food/food-additives-and-gras-ingredients-information-consumers/types-food-ingredients [https://perma.cc/2PQ5-LG56]. *See also* Peter Pressman et al., *Food Additive Safety: A Review of Toxicologic and Regulatory Issues*, Toxicology Rsch. & Application, Mar. 2017, at 1, 5-6, https://journals.sagepub.com/doi/10.1177/2397847317723572 [https://doi.org/10.1177/2397847317723572].

141 21 C.F.R. § 170.38(c)(1)-(4) (2025).

142 21 U.S.C. § 321(t)(1).

143 21 U.S.C. § 379(e).

144
*See* 21 C.F.R. §§ 74, 82 (2025).

145
*See* 21 C.F.R. § 73 (2025).

146 Substances Generally Recognized as Safe, 81 Fed. Reg. 54960, 54962 (Aug. 17, 2016) (codified at 21 C.F.R. pts. 20, 25, 170, 184, 186, 570).

147 21 C.F.R. § 71.1(c) (color additives) (2025); 21 C.F.R. § 171.1(c) (food additives) (2025).

148
*See* sources cited *supra*, note 147.

149
*See* sources cited *supra*, note 147.

150
*See* sources cited *supra*, note 147.

151
*Compare* Redbook 2000, *supra* note 72 (listing genetic toxicity, carcinogenicity, reproductive and neurotoxicity, and others), *with* U.S. Food & Drug Admin., Nonclinical Safety Studies, *supra* note 82 (listing pharmacology, general toxicity, reproductive toxicity, genotoxicity, and carcinogenicity).

152 21 C.F.R. § 70.3(i) (2025). For food additives, the standard is substantially the same: a safe food additive means that “there is a reasonable certainty in the minds of competent scientists that the substance is not harmful under the conditions of its intended use.” 21 C.F.R. 570(i) (2025).

153
*Compare* 21 C.F.R. pt. 314 (2025) (drugs for human use) *with* 21 C.F.R. pt. 171 (2025) (food additive petitions).

154 21 C.F.R. § 312.23(a)(5) (2025).

155 21 C.F.R. §§ 71.1, 171.1 (2025).

156
*See, e.g.*, U.S. Food & Drug Admin., FDA-2013-S-0610, Questions and Answers About the Food Additive or Color Additive Petition, at M-P (2011) (focusing entirely on toxicology and general toxicity).

157 Thomas G. Neltner et al., *Data Gaps in Toxicity Testing of Chemicals Allowed in Food in the United States*, 42 Reprod. Toxicology 85, 88 (2013).

158
*Id.*

159 21 U.S.C. § 355.

160 21 C.F.R. § 71.1(i) (2025).

161 U.S. Food & Drug Admin., 1993 Draft Redbook II at ch. VI (1993).

162 Leonardo Trasande et al., *Food Additives and Child Health*, 142 Am. Ass’n of Pediatrics, Aug. 2018, at 1, 3, https://publications.aap.org/pediatrics/article/142/2/e20181408/37584/Food-Additives-and-Child-Health [https://doi.org/10.1542/peds.2018-1408].

163 Joy Frestedt, *Similarities and Difference between Clinical Trials for Foods and Drugs*, 5 Austin J. Nutrition & Food Sci., June 23, 2017, at 1, 1 https://austinpublishinggroup.com/nutrition-food-sciences/fulltext/ajnfs-v5-id1086.pdf [https://perma.cc/9XBQ-H8LK].

164
*Id.* at 3.

165
*Id.* at 4.

166
*Id.* at 4-5. *See also* Heidi M. Staudacher et al., *The Challenges of Control Groups*, *Placebos and Blinding in Clinical Trials of Dietary Interventions*, 76 Proc. of the Nutr. Soc’y 203, 203 (2017) (explaining the causes of the “paucity of placebo-controlled food and dietary advice trials compared with drug trials”).

167 Hilzenrath, *supra* note 27.

168 Brejt, *supra* note 74, at 47-48 (discussing how individual ingredients can affect how medications interact in the body).

169
*See* 21 U.S.C. § 348(c)(B)(5). *See also* U.K. Committee on Toxicity, Statement on Food Standards for Agency-Funded Research on Health Effects of Mixtures of Food Additives 1 (2008) [hereinafter U.K. Comm. On Toxicity], https://cot.food.gov.uk/sites/default/files/cot/cotstatementmixtures200809.pdf [https://perma.cc/N84C-KBF8] (discussing the cocktail effects of additives).

170
*See supra* Part I.

171
*Food Additives: Hearings Before a Subcomm. of the H. Comm. on Interstate and Foreign Commerce*, 85th Cong. 460-62, 464 (1957) (additional information submitted to the record by Food & Drug Admin).

172 21 U.S.C. § 321(s).

173 Taimie Bryant, *Novel Food Ingredients: Food Safety Law, Animal Testing, and Consumer Perspectives*, 106 Marq. L. Rev. 97, 113 (2022) (discussing manufacturers requests for FDA to publish their substances on GRAS list).

174
*Id.* at 113-14.

175
*Id.*

176 21 C.F.R. § 170.30(a) (2025).

177
*FDA’s Approach to the GRAS Provision: A History of Processes*, U.S. Food & Drug Admin. (Jan. 4, 2018), https://www.fda.gov/food/generally-recognized-safe-gras/fdas-approach-gras-provision-history-processes [https://perma.cc/457U-QVL9].

178
*Id.*

179 Bryant, *supra* note 173, at 115.

180
*Id.*

181 Substances Generally Recognized as Safe, 81 Fed. Reg. 54960 (Aug. 17, 2016) (codified at 21 C.F.R. pts. 20, 25, 170, 184, 186, 570).

182
*See* 21 C.F.R. § 170.30(b) (2025) (same evidence); Gov’t Accountability Off., GAO-10-246, FDA Should Strengthen Its Oversight of Food Ingredients Determined to Be Generally Recognized as Safe (GRAS) 12, 20, 25 (2010), https://www.gao.gov/products/gao-10-246 [https://perma.cc/M8ED-N7Z9] ([C]ompanies are not required to provide information to FDA regarding their GRAS determinations.”).

183 Ctr. for Sci. Pub. Int. et al., Comment Letter on Substances Generally Recognized as Safe (GRAS) Dkt. No. FDA-1997-N-0020, at 15-16, (Apr. 2015), https://www.cspinet.org/sites/default/files/attachment/GRAS%20Comment%20FINAL_0.pdf [https://perma.cc/6XX4-QASX] (private determination and notice). *See* Bryant, *supra* note 173, at 114-15 (discussing proposed voluntary GRAS notification rule); Scrufari, *supra* note 59, at 235-37 (discussing lack of data submission and evasion of pre-market regulatory approval process).

184 Olivia Backhaus & Melanie Benesh, *EWG Analysis: Almost All New Food Chemicals Greenlighted by Industry, Not the FDA*, Env’t Working Grp. (Apr. 13, 2022), https://www.ewg.org/news-insights/news/2022/04/ewg-analysis-almost-all-new-food-chemicals-greenlighted-industry-not-fda [https://perma.cc/9XX2-LLXF].

185
*Id. See also* U.S. Food & Drug Admin., GRAS Notices, https://www.hfpappexternal.fda.gov/scripts/fdcc/index.cfm?set=GRASNotices [https://perma.cc/YL7H-MH8Z] (last visited Oct. 22, 2025).

186 Backhaus & Benesh, *supra* note 184.

187 Neltner et al., *supra* note 157, at 90.

188
*See* National Toxicology Program, Dept of Health & Hum. Servs., Report on Carcinogens: Butylated Hydroxyanisole 1 (2021) (BHA); K. Nadira De Abrew et al., *A New Approach Methodology (NAM) Based Assessment of Butylated Hydroxytoluene (BHT) for Endocrine Disruption Potential*, 190 Toxicol. Sci. 227, 227-28 (2022) (BHT); Sarah Krull Abe & Manami Inoue, *Green Tea and Cancer and Cardiometabolic Diseases: A Review of the Current Epidemiological Evidence*, 75 Eur. J. Clinical Nutrition 865, 865 (2021) (EGCG); Scrufari, *supra* note 59, at 256 (carrageenan).

189
*See* Backhaus & Benesh, *supra* note 184; Scrufari, *supra* note 59, at 256.

190 Scrufari, *supra* note 59, at 242-43. *See also Trans Fats*, U.S. Food & Drug Admin. (Apr. 30, 2024), https://www.fda.gov/food/food-additives-petitions/trans-fat [https://perma.cc/PLZ5-856C].

191
*See* Backhaus & Benesh, *supra* note 184. *See also* Tom Neltner & Maricel Maffini, Nat. Res. Def. Council, *Generally Recognized as Secret: Chemicals Added to Food in the United States* 4 (2014), https://www.nrdc.org/sites/default/files/safety-loophole-for-chemicals-in-food-report.pdf [https://perma.cc/WYH2-AU28].

192 Kimberly Kindy, *Food Additives on the Rise as FDA Scrutiny Wanes*, The Wash. Post (Aug. 17, 2014), https://www.washingtonpost.com/national/food-additives-on-the-rise-as-fda-scrutiny-wanes/2014/08/17/828e9bf8-1cb2-11e4-ab7b-696c295ddfd1_story.html [https://perma.cc/HR5A-VUKG].

193
*See, e.g.*, Coral Beach, *FDA Determines that Tara Flour Is Not Safe; 500 Were Sickened by the Ingredient*, Food Safety News (May 16, 2024), https://www.foodsafetynews.com/2024/05/fda-determines-that-tara-flour-is-not-safe-500-were-sickened-by-the-ingredient/ [https://perma.cc/8C9R-NBG3].

194 U.S. Food & Drug Admin., Regulatory Status and Review of Available Information Pertaining to Tara Protein/flour Derived from the Seed Germ of the Plant, Caesalpinia Spinosa: Lack of General Recognition of Safety for its Use in Foods 8 (2024) [hereinafter FDA Memorandum].

195 Tom Neltner, *Tara Flour: A Reminder of the Real-Life Consequences of Broken GRAS*, Env’t Def. Fund (Aug. 10, 2022), https://blogs.edf.org/health/2022/08/10/tara-flour-a-reminder-of-the-real-life-consequences-of-broken-gras/ [https://perma.cc/JA6D-JKRH]. *See also* Beach, *supra* note 193 (discussing the Peru study).

196
*See* FDA Memorandum, *supra* note 194.

197
*Id.*

198
*See* Redbook 2000, *supra* note 72, at IV.C.6 (“Given the nature of consumption patterns of food ingredients (i.e., chronic, lifetime exposures), it is necessary to require the chronic safety testing of food ingredients that would be representative of lifetime exposure in humans.”).

199
*Color Additives in Food*, U.S. Food & Drug Admin. (July 6, 2023), https://www.fda.gov/food/color-additives-information-consumers/color-additives-foods [https://perma.cc/6DGU-24EW].

200 21 U.S.C. § 343.

201
*Id.*

202 21 C.F.R. § 101.22(k)(2) (2025).

203
*See* Brejt, *supra* note 74.

204 21 C.F.R. § 101.22 (2025).

205 Matthew Goodman, *The “Natural” vs. “Natural Flavors” Conflict in Food Labeling: A Regulatory Viewpoint*, 72 Food Drug L.J. 78, 80 (2017). *See also* Brejt, *supra* note 74.

206 21 C.F.R. § 101.22 (2025).

207 21 C.F.R. § 101.22(h)(2) (2025).

208 Thomas Galligan et al., Hidden Ingredients 11-12 (2024), https://www.cspinet.org/sites/default/files/2024-03/CSPI_FlavorReport2024_FINAL.pdf [https://perma.cc/EY6H-24HH].

209
*See* Brejt, *supra* note 74, at 46.

210
*See* Katya S. Cronin, *Bystanders to a Public Health Crisis: The Failures of the U.S. Multi-Agency Regulatory Approach to Food Safety in the Face of Persistent Organic Pollutants*, 49 Colum. J. Env’t. L. 291, 301-02 (2024) [hereinafter Cronin, *Bystanders*].

211
*Id.*

212 Jennifer L. Pomeranz et al., *Advancing the FDA’s Human Foods Program Through Additional Authorities and User Fees*, 44 Health Affs. 458, 458 (2025); 21 C.F.R. § 80.10(a) (2025).

213 Precedence Research, *Food Additive Market Size and Forecast 2025 to 2034* (May 8, 2025), https://www.precedenceresearch.com/food-additive-market [https://perma.cc/F4LD-YHD3].

214 Stephanie Soucheray, *Analysis: New FDA User Fees May Be Path Forward for Food Safety Funding*, Ctr. for Infectious Disease Rsch .& Pol’y (Apr. 8, 2025), https://www.cidrap.umn.edu/foodborne-disease/analysis-new-fda-user-fees-may-be-path-forward-food-safety-funding [https://perma.cc/WX9A-H86K].

215
*See supra* Part II.

216 Food & Drug Admin., FDA-2020-D-1917 Questions and Answers About the Food Additive or Color Additive Petition Process, at S (2011).

217
*See, e.g.*, 21 C.F.R. § 314.81(b) (2025) (“Other postmarketing reports.”). *See also supra* Part II.

218
*FDA Works to Enhance the Assessment of Ingredients in Foods and Food Contact Substances on the Market*, U.S. Food & Drug Admin. (July 12, 2023), https://www.fda.gov/food/conversations-experts-food-topics/fda-works-enhance-assessment-ingredients-foods-and-food-contact-substances-market [https://perma.cc/Q3NW-XRPS] (“We have the authority to assess the safety of ingredients in foods on the market. However, manufacturers are generally not required to submit post-market use information under our current authorities, so we generally have to rely on less accurate publicly available information or data submitted voluntarily to estimate exposure.”).

219 21 U.S.C. § 350f.

220 Lorne Hofseth, *The FDA Banned Red 3 Food Coloring*, Pub. Broadcasting Station (Jan. 25, 2025), https://www.pbs.org/newshour/health/the-fda-banned-red-3-food-coloring-a-scientist-explains-the-dyes-history-and-health-risks [https://perma.cc/6RRD-266V].

221
*Id.*

222
*Id.*

223
*See* Nicholas Florko, *A 50-year Battle Over One Additive Highlights FDA’s Challenges with Food Safety*, Stat News (Oct. 19, 2023), https://www.statnews.com/2023/10/19/a-50-year-battle-over-one-food-additive-highlights-fdas-challenges-with-food/ [https://perma.cc/M6AW-GQZM].

224 Revocation of Authorization for Use of Brominated Vegetable Oil in Food, 89 Fed. Reg. 55040 (July 3, 2024) (to be codified at 21 C.F.R. pt. 180).

225
*Id.*; Florko, *supra* note 223.

226
*See Regulatory Status of Color Additives*, U.S. Food & Drug Admin., https://hfpappexternal.fda.gov/scripts/fdcc/index.cfm?set=ColorAdditives&sort=Sort_Unique_ID&order=ASC&startrow=51&type=column&search=Use%2Dcurrent%C2%A4VARCHAR%C2%A4foods [https://perma.cc/TSD8-RUAN] (last visited Oct. 6, 2025).

227 Alexandra Hopkins, *What is Food Dye?*, Env’t Working Grp. (Mar. 27, 2024), https://www.ewg.org/news-insights/news/2024/03/what-food-dye [https://perma.cc/LPQ6-2WKP].

228
*See* Miller M.D. et al., *Potential Impacts of Synthetic Food Dyes on Activity and Attention in Children: A Review of the Human and Animal Evidence*, Env’t Health, Apr. 29, 2022, at 1, 7, 16; Rachel M. Rambler et al., *A Review of the Association of Blue Food Coloring With Attention Deficit Hyperactivity Disorder Symptoms in Children*, Cureus, Sep. 16, 2022, at 1, 1.

229 Sarah Kobylewski & Michael F. Jacobson, *Toxicology of Food Dyes*, 18 Int’l J. Occupational Env’t Health 220, 220-34 (2012); *see also* CSPINET, Synthetic Food Dyes and Behavioral Effects in Children: Implications for Regulators, Schools, and Daycare Centers 1, 2 (2021), https://www.cspinet.org/sites/default/files/attachment/Dyes_Fact_sheet_School_Foods_3.8.2021.pdf [https://perma.cc/D2HE-XYLT] (displaying synthetic food dyes may cause or exacerbate neurobehavioral issues in children as demonstrated throughout study).

230 CSPINET, *supra* note 229, at 2; Kobylewski & Jacobson, *supra* note 229, at 221.

231 Rambler, *supra* note 228, at 1.

232
*See id.* at 2*; See also id.* at 1, 4-5 (displaying Blue No.1 and analogs possibly impact brain’s susceptibility to metal toxicity).

233 Kobylewski & Jacobson, *supra* note 229, at 221; *See also Public Health Statement Benzidine*, ATSDR 1, 3 (2001), https://www.atsdr.cdc.gov/ToxProfiles/tp62-c1-b.pdf [https://perma.cc/M7BF-RMC2] (holding findings display carcinogens have been found in dyes to color foods).

234 Kobylewski & Jacobson, *supra* note 229, at 221.

235 David B. Weisbrod et al., *A Case Report of Allergic Hypersensitivity to Color Additives in Slurpee Beverages*, Yale J. Biology Med. 79, 79-82 (2023).

236
*See* Sara Jarmakiewicz-Czaja et al., *The Impact of Selected Food Additives on Gastrointestinal Tract in the Example of Nonspecific Inflammatory Bowel Disease*, 5 Archives Med. Sci. 1286, 1290-93 (2022). *See also* Iris Myers, *Public Health Groups Urge FDA to Cancel Approval of Titanium Dioxide in Food*, Env’t. Working Grp. (May 8, 2023), https://www.ewg.org/news-insights/news-release/2023/05/public-health-groups-urge-fda-cancel-approval-titanium-dioxide [https://perma.cc/B6NA-WAAN] (holding the European Union banned titanium dioxide and the United States should act similarly).

237
*See* Martin Hahn & Rebecca Popkin, *HHS and FDA Announce Plans to Phase Out Certified Colors*, Hogan Lovells (Apr. 29, 2025), https://www.hoganlovells.com/en/publications/hhs-and-fda-announce-plans-to-phase-out-certified-colors [https://perma.cc/5CR8-63ER].

238 Kobylewski & Jacobson, *supra* note 229, at 222, 244.

239 Miller M.D. et al., *supra* note 228, at 16. *See also* Roxana R. Soroudi, *Government Repudiation of Americans’ Safety: A Call for Reformulation of FDA’s GRAS Notification Program*, 75 Food & Drug L.J. 39, 63 (2020) (holding Western diet has a multitude of processed foods harming human health).

240 Soroudi, *supra* note 239, at 39, 63.

241
*See, e.g.*, Sari Lehto et al., *Comparison of Food Colour Regulations in the EU and the US: A Review of Current Provisions*, 34 Food Additives & Contaminants 335, 338 (2017) (Orange B, Citrus Red No. 2 and FD&C Green No. 3 banned in the EU); *The Ingredients banned in the EU*, *but legal in the US*, ISITCLEAN (June 17, 2025), https://isitclean.org/the-ingredients-banned-in-the-eu-but-legal-in-the-us/#:~:text=Several%20artificial%20food%20colorings%20that,The%20safety%20of%20Yellow%20No [https://perma.cc/MR78-NPCH] (Blue 1, Blue 2, Green 3, Red 40, Yellow 5, and Yellow 6 banned in the EU); Mikaela Conley, *Titanium Dioxide, Banned in Europe, Is One of the Most Common Food Additives in the U.S.*, U.S. Right to Know (May 12, 2025), https://usrtk.org/chemicals/titanium-dioxide/#:~:text=EU%20ban%20on%20titanium%20dioxide,eliminate%20concerns%20about%20its%20genotoxicity [https://perma.cc/U6MB-QN9H].

242 Trasande et al., *supra* note 162, at 6.

243 Aila Slisco, *The Most Widely Banned Food Dyes—And Why They’re Bad for You*, Newsweek (Dec. 13, 2024), https://www.newsweek.com/most-widely-banned-food-dyes-why-theyre-bad-you-2000207#:~:text=Newsweek%20reached%20out%20for%20comment,many%20other%20countries%2C%20including%20Canada [https://perma.cc/F9T4-CSNW].

244
*FDA Updates the Everything Added to Food in the U.S. Inventory*, U.S. Food & Drug Admin. (June 26, 2018), https://www.fda.gov/food/hfp-constituent-updates/fda-updates-everything-added-food-us-inventory [https://perma.cc/3YEL-N2XJ].

245 Hilzenrath, *supra* note 27.

246 Trasande et al., *supra* note 162, at 7.

247 Jarmakiewicz-Czaja et al., *supra* note 236, at 1290.

248
*Id.* at 1289-93.

249
*Id.* at 1293

250 Hilzenrath, *supra* note 27.

251
*Id.*

252
*Scores of Baked Goods Contain Possible Cancer-Causing Additive*, Env’t Working Grp. (Oct. 14, 2015), https://www.ewg.org/news-insights/news-release/scores-baked-goods-contain-possible-cancer-causing-additive [https://perma.cc/L2GZ-6NYX].

253 Revocation of Authorization for Use of Brominated Vegetable Oil in Food, 89 Fed. Reg. 55040 (July 3, 2024) (to be codified at 21 C.F.R. pt. 180); *FDA Removes 7 Synthetic Flavoring Substances from Food Additives List*, U.S. Food & Drug Admin. (Oct. 5, 2018), https://www.fda.gov/food/hfp-constituent-updates/fda-removes-7-synthetic-flavoring-substances-food-additives-list [https://perma.cc/2W4Q-PL9G] (discussing the revocation of benzophenone, ethyl acrylate, eugenyl methyl ether (methyl eugenol), myrcene, pulegone, pyridine, and styrene).

254 21 U.S.C. § 348(h)(6).

255 Jerome H. Heckman & Deborah W. Ziffer, *Fathoming Food Packaging Regulation Revisited*, 56 Food Drug L.J. 179, 179 (2001). For substances of higher concern, the FDA could still require a food additive petition for an FCS today. 21 U.S.C. § 348(b).

256 21 C.F.R. § 170.39 (2025).

257 Food and Drug Administration Modernization Act of 1997, Pub. L. No. 105-115, 105 Stat. 1677 (1997).

258 21 U.S.C. § 348(h).

259 21 U.S.C. § 348(h)(3).

260
*Authorized Uses of PFAS in Food Contact Applications*, U.S. Food & Drug Admin. (Jan. 3, 2025) https://www.fda.gov/food/chemical-contaminants-food/authorized-uses-pfas-food-contact-applications [https://perma.cc/9MF9-AQ8Y] [hereinafter *PFAS Uses*].

261 21 C.F.R. § 170.3(i) (2025).

262 21 C.F.R. § 170.104 (2025). *See* Katya S. Cronin, *FDA-Approved: How PFAS-Laden Food Contact Substances Are Poisoning Consumers and What to Do About It*, 6 Bus. Entrepreneurship & Tax L. Rev. 117, 133-34 (2022) [hereinafter Cronin, *FDA-Approved*].

263
*See* U.S. Food & Drug Admin., Preparation of Food Contact Substance Notifications (Toxicology Recommendations): Guidance for Industry (2021). *See also* U.S. Food & Drug Admin., Guidance for Industry: Preparation of Premarket Submissions for Food Contact Substances (Chemistry Recommendations) (2007) (outlining calculations for estimating dietary exposures to components).

264 Heckman & Ziffer, *supra* note 255, at 181.

265
*See* Cronin, *FDA-Approved*, *supra* note 262, at 133-35.

266
*FDA Proposes Changes to FCN Program*, Bergeson & Campbell, P.C. (Jan. 27, 2022), https://www.lawbc.com/fda-proposes-changes-to-fcn-program/#:~:text=Commentary,FCN%20is%20no%20longer%20effective [https://perma.cc/W3FS-5PHW].

267 U.S. Food & Drug Admin., Preparation of Food Contact Substance Notifications (Administrative): Guidance for Industry 4-5 (2021).

268 Heckman & Ziffer, *supra* note 255, at 179.

269
*See* Cronin, *FDA-Approved*, *supra* note 262, at 135-37.

270
*See, e.g.*, *id.*; *see also FDA Determines Authorization for 35 Food Contact Notifications Related to PFAS Are No Longer Effective*, U.S. Food & Drug Admin. (Jan. 3, 2025) https://www.fda.gov/food/hfp-constituent-updates/fda-determines-authorization-35-food-contact-notifications-related-pfas-are-no-longer-effective#:~:text=In%20July%202020%2C%20manufacturers%20or%20suppliers%20of,food%20contact%20use%20in%20the%20U.S.%20market [https://perma.cc/5TSJ-BJM2] [hereinafter *FDA PFAS Notice*] (noting that “these 35 FCNs have been abandoned because the manufacturers or suppliers have ceased production, supply, or use of the food contact substances” after companies “voluntarily agreed to phase-out their sales of the grease-proofing substances that contained PFAS.”).

271
*See, e.g.*, Rakesh Kumar Gupta et al., *Migration of Chemical Compounds from Packaging Materials into Packaged Foods: Interaction, Mechanism, Assessment, and Regulations*, 13 Foods 3125, 9, 12, 19 (2024); *see also* Jane Muncke et al., *Impacts of Food Contact Chemicals on Human Health: A Consensus Statement*, 19 Env’t Health 1, 3 (2020) (discussing the lack of testing of hazard properties of the chemicals intentionally used in the manufacturing of food contact articles).

272
*See, e.g.*, Trasande et al., *supra* note 162, at 2-6.

273
*See id.*

274
*Id.* at 3.

275
*Id.* at 7.

276
*See* Cronin, *FDA-Approved*, *supra* note 262, at 135-36 (detailing how FDA regulation for short-chain FCS is based on individual compound toxicity).

277
*See id.* at 123-24; *see also FDA PFAS Notice*, *supra* note 270, at 1.

278
*See PFAS Uses*, *supra* note 260, at 2-3; Shiwen Li et al., Associations Between Per-and Polyfluoroalkyl Substances (PFAS) and County-level Cancer Incidence between 2016 and 2021 and Incident Cancer Burden Attributable to PFAS in Drinking Water in the United States, 35 J. Expo. Sci. Environ. Epidemiol. 425, 425 (2025) (“We found that PFAS in drinking water was associated with cancers in the organ system including the oral cavity/pharynx, lung, digestive system, brain, urinary system, soft tissue, and thyroid.”).

279 Trasande et al., *supra* note 162, at 3.

280
*See id.* (explaining how lack of FDA regulation for diisodecyl and diisononylphthalate has increased its prevalence in urinary metabolites of humans).

281
*See generally* Cronin, *One-Health*, *supra* note 50 (detailing prevalence of environmental contaminants in various forms).

282
*See generally* Cronin, *Bystanders*, *supra* note 210 (discussing limits to F.D.A. enforcement powers due to E.P.A. and U.S. Department of Agriculture jurisdiction).

283 Food Safety Modernization Act, Pub. L. No. 111-353, § 106, 124 Stat. 3905, 3926-27 (2011).

284 21 U.S.C. § 350l.

285 21 U.S.C. § 334(h)(1)(A).

286 21 U.S.C. §§ 381, 384d.

287 21 U.S.C. § 350j(a)(2).

288
*See FSMA Compliance: Get ready for FSMA 204*, Telus Agric. & Consumer Goods (Jan. 3, 2025), https://www.telus.com/agcg/en/blog-resources/fsma-compliance [https://perma.cc/7MTD-ZCG7].

289 21 C.F.R. § 109.6 (2025).

290
*See id.* (stating that the enforcement powers for tolerances, regulatory limits, and action limits rests with the F.D.A).

291 21 C.F.R. § 165.110 (2025).

292 U.S. Food & Drug Admin., Compliance Policy Guide Sec. 555.400 Aflatoxins in Human Food: Guidance for FDA Staff 2 (2021).

293
*See Chemical Contaminants*, U.S. Food & Drug Admin. (June 17, 2025) https://www.fda.gov/animal-veterinary/biological-chemical-and-physical-contaminants-animal-food/chemical-contaminants [https://perma.cc/4PLW-KMNC] (noting no tolerance for PFAS and dioxins, and only temporary tolerances for PCBs in animal feed); *Supporting Document for Guidance Levels for Radionuclides in Domestic and Imported Foods*, U.S. Food & Drug Admin. (Feb. 25, 2025), https://www.fda.gov/food/process-contaminants-food/supporting-document-guidance-levels-radionuclides-domestic-and-imported-foods (providing guidance but no binding tolerances for radionuclides); *Microplastics and Nanoplastics in Foods*, U.S. Food & Drug Admin. (July 24, 2025), https://www.fda.gov/food/environmental-contaminants-food/microplastics-and-nanoplastics-foods (noting that FDA does not currently regulate microplastics and nanoplastics in food).

294
*Environmental Contaminants in Food*, U.S. Food & Drug Admin. (Jan. 17, 2025), https://www.fda.gov/food/chemical-contaminants-pesticides/environmental-contaminants-food [https://perma.cc/YB2B-9UMV].

295 U.S. Food & Drug Admin., Detention without Physical Examination of Foods Due to Chemical Contamination: Import Alert 99-48 (2025).

296 U.S. Food & Drug Admin., Control of Listeria monocytogenes in Ready-To-Eat Foods: Guidance for Industry 3-6 (2017).

297
*Closer to Zero: Reducing Childhood Exposure to Contaminants from Foods*, U.S. Food & Drug Admin. (Jan. 6, 2025), https://www.fda.gov/food/environmental-contaminants-food/closer-zero-reducing-childhood-exposure-contaminants-foods [https://perma.cc/N8CS-RR48].

298
*See, e.g.*, Scott Faber, *How Factory Farms Can Contaminate Carrots with Dangerous Pathogens Like E. Coli*, Env’t Working Grp. (Nov. 19, 2024), https://www.ewg.org/news-insights/news/2024/11/how-factory-farms-can-contaminate-carrots-dangerous-pathogens-e-coli [https://perma.cc/9WFK-WYC2] (detailing recalls for carrots in California due to an E. Coli outbreak).

299
*What to Know About Heavy Metals in Baby Food*, Cleveland Clinic (June 20, 2024), https://health.clevelandclinic.org/should-parents-be-worried-about-toxic-heavy-metals-in-baby-food [https://perma.cc/ZPG2-47DT] (emphasis added); *see also Investigation of Elevated Lead & Chromium Levels: Cinnamon Applesauce Pouches*, U.S. Food & Drug Admin. (Aug. 15, 2024), https://www.fda.gov/food/outbreaks-foodborne-illness/investigation-elevated-lead-chromium-levels-cinnamon-applesauce-pouches-november-2023 [https://perma.cc/SNK9-D37Z]; Patrick Gray, *A Survey of Toxic Elements in Ready to Eat Baby Foods in the U.S. Market 2021*, 16 Food Additives & Containments: Part B 79, 80-81 (2023) (finding heavy metals in 386 of 400 samples baby foods).

300 Dorothy Atkins, *Amazon.com Sued Over Toxic Metals Found in Rice Products*, Law360 (May 23, 2025), https://www-law360-com.ezproxy.bu.edu/articles/2344611/print?section=classaction [https://perma.cc/M2MP-HTNC].

301
*See, e.g.*, Metz, *supra* note 26, at 381 (detailing a proposed solution to improve public health by improving food quality and accessibility of healthy options); *See, e.g.*, Sanders, *supra* note 27 (describing support across Americans restricting ingredients in processed foods to improve public health).

302
*See, e.g.*, Evich, *supra* note 29 (describing grassroots support for RFK Jr’s ‘Make America Healthy Again’ agenda).

303
*See generally* Gov’t Accountability Off., GAO-25-107571, Food Safety: FDA Should Strengthen Inspection Efforts to Protect the U.S. Food Supply (2025) (describing how the F.D.A. should improve inspection efforts to meet governmental requirements and ensure food safety); Howard Sklamberg, *Improving the FDA’s Food Safety Program: Choosing Practical Solutions*, Think Glob. Health (Aug. 13, 2022), https://www.thinkglobalhealth.org/article/improving-fdas-food-safety-program-choosing-practical-solutions [https://perma.cc/VLD9-A8P6] (detailing multiple proposals for revising F.D.A. food safety policy).

304
*See* Moira Warburton, *Why Congress Is Becoming Less Productive*, Reuters (Mar. 12, 2024), https://www.reuters.com/graphics/USA-CONGRESS/PRODUCTIVITY/egpbabmkwvq/ [https://perma.cc/EW7K-QSWW] (visualizing decrease of bills passed over time).

305
*See* Evich, *supra* note 29.

306 Tin, *supra* note 30.

307
*See* Metz, *supra* note 26; Sanders, *supra* note 27; Orth, *supra* note 27; Hilzenrath, *supra* note 27.

308
*See, e.g.*, Scrufari, *supra* note 59, at 237 (stating that the GRAS scheme has created “a giant backdoor - a loophole of epic proportions”).

309
*See Pre-1938 Drugs Not Approved by FDA*, Int’l J. of Pharma. Compounding: CompoundingToday.Com, https://compoundingtoday.com/Compliance/FDAPre1938Drugs.cfm#:~:text=In%201938%2C%20a%20bill%20was,Drug%20Products%20and%20Legal%20Requirements [https://perma.cc/F9U6-E5NV] (last visited June 19, 2025).

310 Drug Amendments of 1962, Pub. L. 87-781, 76 Stat. 780 (amending 21 U.S.C. §§ 301-399).

311
*Drug Efficacy Study Implementation (DESI)*, U.S. Food & Drug Admin. (May 16, 2024), https://www.fda.gov/drugs/enforcement-activities-fda/drug-efficacy-study-implementation-desi [https://perma.cc/S2MX-D7FU].

312
*See* Michelle Meadows, *Promoting Safe & Effective Drugs for 100 Years*, FDA Consumer, Mag. Jan.-Feb. 2006, at 15, 17.

313
*See* Matej Mikulic, *Global Pharmaceutical Industry - Statistics & Facts*, Statista (Nov. 22, 2024), https://www.statista.com/topics/1764/global-pharmaceutical-industry/ [https://perma.cc/8QJF-H4M8] (“The global pharmaceutical industry has experienced significant growth during the past two decades, with revenues totaling around 1.6 trillion U.S. dollars in 2023.”).

314
*See, e.g.*, Deborah Blum, The Poison Squad: One Chemist’s Single-Minded Crusade for Food Safety at the Turn of the Twentieth Century 2-3 (2018) (detailing the many dangerous additives, including formaldehyde, which were used in food prior to 1958).

315
*See, e.g.*, Interstate Tech. Regul. Council, History and Use of Per- and Polyfluoroalkyl Substances (PFAS) Found in the Environment 1-2 (2020), https://pfas-1.itrcweb.org/wp-content/uploads/2020/10/history_and_use_508_2020Aug_Final.pdf [https://perma.cc/VKD7-QZXR] (noting that PFAS were chemically invented in 1930 and commercially available in many consumer products since 1950).

316
*See* Redbook 2000, *supra* note 72, at IV.C.6.

317
*Id.*; *See, e.g.*, Trasande et al., *supra* note 162, at 2 (listing the many food additives that have been proven harmful to human health).

318
*See* Cronin, *FDA-Approved*, *supra* note 262, at 127-29 (discussing the bioaccumulation and bio-persistence of PFAS).

319
*See* Kapczynski, *supra* note 78, at 2362-63.

320
*See* Soroudi, *supra* note 239, at 53 (noting that industry currently uses GRAS designations for novel and untested substances).

321
*GRAS Notices*, U.S. Food & Drug Admin., https://www.hfpappexternal.fda.gov/scripts/fdcc/index.cfm?set=GRASNotices [https://perma.cc/JMJ2-PQ69] (last visited Sep. 10, 2025).

322 Maria Doa, Liora Fiksel & Maricel Maffini, *Six Ways FDA Can Do Better on Food Safety*, Env’t Def. Fund (Feb. 6, 2025), https://blogs.edf.org/health/2025/02/06/six-ways-fda-can-do-better-on-food-safety/ [https://perma.cc/FEG4-FCEU] (discussing the need for comprehensive rather than focused safety reevaluation).

323
*See* 21 U.S.C. § 346a(q) (requiring re-assessment of pesticides in a graduated manner: thirty-three percent within three years, sixty-six percent within six, and one-hundred percent within ten years).

324
*See* 21 U.S.C. § 355(b)(1)(A)(i-vi).

325
*See* Soroudi, *supra* note 239, at 51.

326
*See, e.g.*, Soroudi, *supra* note 239, at 6.

327
*See* 21 C.F.R. § 312.22 (2025).

328 The Flavor and Extract Manufacturers Association, for example, states that it conducts all necessary and published all its safety data on GRAS determinations in peer reviewed literature. *See About FEMA GRAS Program*, FEMA, https://www.femaflavor.org/gras [https://perma.cc/E4MQ-KWAM] (last visited Sep. 10, 2025).

329
*See* 21 U.S.C. § 342(a)(2)(C)(i); 21 C.F.R. § 170.30(g) (2025).

330
*See, e.g.*, *GRAS: What It Is and How It Benefits Manufacturers and Consumers*, Axiom Foods (Oct. 4, 2019), https://axiomfoods.com/fda-gras-what-it-is-how-benefits-manufacturers-and-consumers/ [https://perma.cc/B7NM-SFX4] (“Additionally, it is more burdensome on manufacturers to use food additives versus food GRAS.”).

331
*See, e.g.*, Becerra, 565 F. Supp. 3d at 537 (noting that proponents of GRAS argue that “[t]he GRAS Notification program allows FDA to best direct these limited resources” and “a change to the GRAS Rule would result in confusion and overwhelm FDA with reviewing the status of countless ingredients that are being used in food.”).

332
*What We Do*, U.S. Food & Drug Admin. (Nov. 21, 2023), https://www.fda.gov/about-fda/what-we-do [https://perma.cc/KH55-FHFG] (“The Food and Drug Administration is responsible for protecting the public health by ensuring the safety, efficacy, and security of human and veterinary drugs, biological products, and medical devices; and by ensuring the safety of our nation’s food supply, cosmetics, and products that emit radiation.”).

333
*See, e.g.*, Kimberly Kindy, *Food Additives on the Rise as FDA Scrutiny Wanes*, The Wash. Post (Aug. 17, 2014), https://www.washingtonpost.com/national/food-additives-on-the-rise-as-fda-scrutiny-wanes/2014/08/17/828e9bf8-1cb2-11e4-ab7b-696c295ddfd1_story.html [https://perma.cc/F28T-PS8F].

334 Hilzenrath, *supra* note 27.

335
*See, e.g.*, 21 U.S.C. § 346a(q) (the Food Quality Protection Act amended the FDCA to require more comprehensive safety inspections, but introduced tiered levels of review, graduated quotas, and tolerances).

336
*See, e.g.*, Sara Smith, *Why the United States Should Change its Standards for Food Additives*, 39 Touro L. Rev. 1519, 1545-46 (2024); *Regulatory Challenges of New Foods: EU and US Perspective*, ChemSafe Blog (Sep. 11, 2025, at 04:13 ET), https://www.chemsafe-consulting.com/en/2024/10/15/regulatory-challenges-of-new-foods-eu-and-us-perspective/ [https://perma.cc/G96T-AUEP] (“In the EU, there is nothing like GRAS status; there is only traditional vs novel food.”).

337
*See, e.g.*, Vani Hari, *Food in America compared to the U.K. (Why is it so different?)*, Food Babe (Sep. 11, 2025, at 04:15 ET), https://foodbabe.com/food-in-america-compared-to-the-u-k-why-is-it-so-different [https://perma.cc/HH3E-EGWK] (comparing ingredients of products in the US and UK).

338 21 U.S.C. § 348(h).

339
*See generally* Cronin, *FDA-Approved*, *supra* note 262 (discussing how PFAS were “approved” for use in food contact materials and the difficulties in revoking those food contact notices).

340
*See generally* Trasande et al., *supra* note 162 (exploring the negative health consequences from indirect food additives in food contact materials); Cronin, *FDA-Approved*, *supra* note 262 (reviewing sources on the negative health effects of PFAS in food contact materials).

341
*See* 21 U.S.C. § 348(h)(3).

342 21 U.S.C. § 348(c)(3)(A).

343 Scrufari, *supra* note 59, at 244-45.

344
*Id.*

345 Noah & Merrill, *supra* note 14, at 395.

346 Scrufari, *supra* note 59, at 243-51.

347
*See* Monsanto Co. v. Kennedy, 613 F.2d 947, 955 (D.C. Cir. 1979).

348
*See* Scrufari, *supra* note 59, at 243-51.

349
*See id.; Compare* Scott v. Food and Drug Admin., 728 F.2d 322, 325 (6th Cir. 1984) (finding a de minimis exception for food contact substances), *with* Public Citizen v. Young, 831 F.2d 1108 (D.C. Cir. 1987) (finding no de minimis exception for color additives).

350
*See* Noah & Merrill, *supra* note 14, at 395.

351
*See, e.g.*, Tayebeh Soltanighias et al., *Combined Toxicity of Perfluoroalkyl Substances and Microplastics on the Sentinel Species Daphnia Magna: Implications for Freshwater Ecosystems*, 363 Env’t Pollution 125133, at 8 (2024) (describing the “synergistic and additive effects” of microplastics and PFAS in food); Marie Payen de la Garanderie et al., *Food Additive Mixtures and Type 2 Diabetes Incidence: Results from the NutriNet-Santé Prospective Cohort*, 22 Observ. Study 1, 1 (2025) (finding compounding effects on Type 2 diabetes occurrence of two common food additive mixtures and noting that “a combination of food additives may be of interest to consider in safety assessments, and they support public health recommendations to limit nonessential additives.”); Cynthia Recoules et al., *Evaluation of the Toxic Effects of Food Additives, Alone or in Mixture, in Four Human Cell Models*, 196 Food and Chem. Toxic. 1, 10 (2025) (finding that two tested mixtures induced genotoxicity that “could not be attributed to the relative concentrations of the individual additive” and noting “the limitations of risk assessments based solely on individual compound effects and [] the importance of evaluating chemical mixtures.”).

352
*See* Gov’t Accountability Off., RCED 93-142, Food Safety and Quality: Innovative Strategies May Be Needed to Regulate New Food Technologies 89-95 (1993); *see also* Soroudi, *supra* note 239, at 63 (proposing a mandatory GRAS notification program as a potential strategy for statutory reform).

353
*See* Pomeranz et al., *supra* note 212, at 458.

354 Lisa Held, *Cuts at FDA Include Staff Working on Food Safety*, Civil Eats (Feb. 19, 2025), https://civileats.com/2025/02/19/cuts-at-fda-include-staff-working-on-food-safety/ [https://perma.cc/4ASF-UB5L].

355
*See* Consumer Fin. Protection Bureau v. Cmty Fin. Servs. Ass’n of Am., 601 U.S. 416, 421 (2024).

356 Helena Bottemiller Evich, *Trump’s Budget Seeks Billions in Cuts, But More MAHA Money*, FoodFix (June 3, 2025), https://foodfix.co/trumps-budget-seeks-billions-in-cuts-but-more-maha-money/ [https://perma.cc/YC6Z-98CH].

357 Pomeranz et al., *supra* note 212, at 462.

358
*FDA: User Fees Explained*, U.S. Food & Drug Admin. (May 22, 2024), https://www.fda.gov/industry/fda-user-fee-programs/fda-user-fees-explained [https://perma.cc/Y96H-TWWD].

359 Pomeranz et al., *supra* note 212, at 462.

360 Prescription Drug User Fee Act of 1992, Pub. L. No. 102-571, 106 Stat. 4491; Generic Drug User Fee Amend. of 2012, Pub. L. No. 112-144, 126 Stat. 1008; Food Safety Modernization Act, Pub. L. No. 111-353, §§ 107, 306-07, 124 Stat. 3885, 3906, 3958-66 (2011).

361 Aaron, *The Fall of FDA Review*, *supra* note 132, at 153-55, 157, 181, 184.

362
*See* Erika Lietzan, *User Fee Programs*, 76 Admin. L. Rev. 375, 376-77 (2024) (Noting that “hundreds of user fees are in place across dozens of agencies” and that “[s]ome agencies are fully funded by user fees, some agencies have discrete programs that are fully funded by user fees, and some agencies receive only modest support from user fees.”).

363
*See, e.g.*, 15 Yuma C. of Ordinances, § 157-01 (“The payment of development fees does not entitle the applicant to a construction permit unless all applicable land use, zoning, planning, platting, subdivision, and other related requirements, standards, and conditions have been met. Such other requirements, standards, and conditions are independent of the requirement for payment of development fees.”); *Filing Fees*, U.S. Citizenship & Immigr. Serv. (Oct. 28, 2025), https://www.uscis.gov/forms/filing-fees [https://perma.cc/XD38-6JQQ] (“Filing and biometric service fees are final and nonrefundable, regardless of any action we take on your application, petition, or request, or if you withdraw your request.”); Ga. Code § 40-5-25(b)(1) (“If said person fails to achieve a passing score on the knowledge test, the license fee paid shall be considered a testing fee and retained by the department. Any person failing to achieve a passing score on the knowledge test for an instruction permit shall pay the applicable license fee on each subsequent attempt until successful, at which time said fee shall be his or her license fee.”).

364
*See, e.g.*, Courtney Begley, *“So Close, Yet So Far”: The United States Follows the Lead of the European Union in Mandating GMO labeling. But Did It Go Far Enough?*, 40 Fordham Int’l L.J. 625, 630 (2017) (“The FDA relies solely on voluntary information provided by food manufacturers to ensure safety and compliance with its standards.”); *FDA PFAS Notice*, *supra* note 270 (noting voluntary industry phase outs of 35 PFAS chemicals from grease-proof paper products).

365 Soroudi, *supra* note 239, at 52.

366
*See, e.g.*, Inst. of Med. & Nat’l Rsch. Council, Dietary Supplements: A Framework for Evaluating Safety 164 (2005) (“As with any type of scientific study in which an effect is not observed, it is important to remember that a lack of observed or reported detrimental effects in an animal study is not adequate evidence that a particular substance is ‘safe’ to humans.”); *Food Additives and IBD*, Nutrition & Lifestyle Med. Clinic, https://nalmclinic.com/blog-1/2021/3/8/food-additives-and-ibd [https://perma.cc/46JF-CQD9] (last visited Sep. 28, 2025) (“It’s important to note that, at the moment, the evidence we have with regards to food additives is mainly in animal studies with only a handful of small human trials. Whilst animal trials can provide interesting information and inform direction of further research in humans, we cannot take animal trial findings as gospel as sometimes the findings in humans differ as we are different species.”).

367 Hilzenrath, *supra* note 27.

368 Neltner et al., *supra* note 157, at 88.

369
*Cf. supra* Part II.

370
*See, e.g.*, McCarthy, *supra* note 10; Trasande et al., *supra* note 162, at 6-7; Andrea Petersen, *Food Additives Could Combine to Threaten Health, Study Says*, Wall St. J., (Apr. 24, 2025), https://www.wsj.com/health/wellness/food-dyes-sweeteners-emulsifiers-health-study-2e7ca896?st=e5sxfj&reflink=desktopwebshare_permalink [https://perma.cc/L4N7-ZXWA].

371
*See* Redbook 2000, *supra* note 72, at 142-53; Maricel Maffini & Thomas Neltner, *Brain Drain: The Cost of Neglected Responsibilities in Evaluating Cumulative Effects of Environmental Chemicals*, 69 J. Epidemiology & Cmty. Health 496, 496-99 (2015).

372
*See generally* Kapczynski, *supra* note 78 (discussing FDA’s information production function for pharmaceutical review).

373
*See* Doa, Fiksel & Maffini, *supra* note 325 (emphasizing that though comprehensive assessments and the formation of a scientific advisory committee, the “FDA can take immediate action on priority chemicals” while also working to combat the introduction of new harmful ingredients on the market). For comparison, it takes a new food product around 2 years to pass safety review and be introduced on the market. *See Regulatory Challenges of New Foods: EU and US Perspective*, *supra* note 336.

374
*See, e.g.*, Doa, Kiksel & Maffini, *supra* note 322 (arguing that FDA should conduct comprehensive review and save focused assessments only for emergent situations).

375
*See* 21 U.S.C. § 350f.

376
*See* 21 U.S.C. § 355(k) (allowing FDA to require reports and records from drug manufacturers); 21 C.F.R. § 314.81(b)(2) (2025) (requiring annual reports).

377
*See* Neltner et al., *supra* note 157, at 92 (discussing the fact that other agencies, like EPA, have authorizations to track annual chemical usage, while the FDA does not).

378
*How to Report a Problem with Dietary Supplements*, U.S. Food & Drug Admin. (Mar. 13, 2025), http://www.fda.gov/Food/DietarySupplements/ReportAdverseEvent/ [https://perma.cc/BA6P-PXVU]; *FDA Adverse Event Reporting System (FAERS) Database*, U.S. Food & Drug Admin. (Dec. 7, 2023), https://www.fda.gov/drugs/fdas-adverse-event-reporting-system-faers/fda-adverse-event-reporting-system-faers-public-dashboard [https://perma.cc/97D8-V6PJ]; *Reportable Food Registry for Industry*, U.S. Food & Drug Admin. (Mar. 28, 2022), https://www.fda.gov/food/compliance-enforcement-food/reportable-food-registry-industry [https://perma.cc/7773-YM82].

379 Scrufari, *supra* note 59, at 239 (advocating for a database of consumer reportable events).

380
*See* 21 U.S.C. § 350f(a)(2).

381
*See, e.g.*, Lauren Kirchner, *Forever Chemicals’ Are Found in Some Milk, Including Organic*, Consumer Reps. (May 2, 2024), https://www.consumerreports.org/pfas/pfas-forever-chemicals-found-in-some-milk-including-organic-a1101576034/ [perma.cc/XK7P-YTCN] (listing the results of Consumer Report independent testing of PFAS in 50 milk samples); Leah Segedie, *Cinnamon Tested for Glyphosate*, *Lead*, *& Cadmium — Buying Guide*, Mamavation (Apr. 23, 2024), https://mamavation.com/food/cinnamon-lead-cadmium-glyphosate.html [perma.cc/8R4D-2PZH] (listing results from private testing of cinnamon brands for glyphosate, lead, and cadmium).

382
*See, e.g.*, Atkins, *supra* note 300 (discussing class action against Amazon over heavy metals in rice products); PFAS Citizens Petition from Env’t Def. Fund et al., to U.S. Food & Drug Admin. (June 3, 2021), https://blogs.edf.org/health/wp-content/blogs.dir/11/files/2021/06/PFAS-Petition-to-FDA-FINAL-6-1-21.pdf [https://perma.cc/9LS8-N7JU] (“Requesting that the agency take more aggressive action to protect consumers from per- and poly-fluoroalkyl substances (PFAS) by banning all forms that biopersist in the human body.”).

383
*See* U.S. Gov’t Accountability Off., *supra* note 303, at 10 (“FDA officials identified limited workforce capacity as FDA’s primary challenge to meeting inspection targets.”).

384 Press Release, U.S. Dep’t. of Health & Hum. Servs., *HHS Secretary Kennedy Directs FDA to Explore Rulemaking to Eliminate Pathway for Companies to Self-Affirm Food Ingredients Are Safe* (Mar. 10, 2025), https://www.hhs.gov/press-room/revising-gras-pathway.html [https://perma.cc/JGB4-R6VZ].

385
*See* Memorandum from Avia Dunn & Rachel Turow to Skadden, Arps. Slate, Meagher & Flom LLP affiliates (Mar. 13, 2025), https://www.skadden.com/insights/publications/2025/03/closing-the-gras-loophole-secretary-kennedys-plan-to-enhance-fda-food-safety-regulations [https://perma.cc/3Z7T-NR43] (discussing the implications of making GRAS notices mandatory).

386
*What is the FDA Doing to Reduce the Diabetes and Obesity Epidemics in America and Take on the Greed of the Food and Beverage Industry? Hearing of the S. Comm. on Health, Educ., Lab., & Pensions*, 118th Cong. 22 (2024) (statement of Robert Califf, Comm’r, U.S. Food & Drug Admin).

387
*Cf.* Statement of Policy: Foods Derived from New Plant Varieties, 57 Fed. Reg. 22984, 22990 (May 29, 1992) (FDA stated its intention to presume that foods produced through the rDNA process were “generally recognized as safe”).

388
*See* 21 C.F.R. § 170.265(b) (2025) (explaining that FDA can act within 180 days or take an indeterminate number of ninety-day extensions).

389 Doa, Fiksel & Maffini, *supra* note 322.

390
*See* Lixia Fu et al., *The Applications and Advances of Artificial Intelligence in Drug Regulation: A Global Perspective*, 15 Acta Pharm. Sinica B. 1, 11 (2025) (“Additionally, AI can be fully utilized to conduct regulatory scientific research. This includes assessing drug safety in specific populations, establishing regulatory standards for future new technology treatment products, and analyzing RWD, to facilitate the rapid transformation and application of novel therapeutics.”).

391
*See* 21 C.F.R. § 170.30(b) (2025) (GRAS standard for approval based on scientific procedures the same as petition standard).

392
*See* U.S. Gov’t Accountability Off., GAO/RCED-92-142, Food Safety & Quality: Innovative Strategies May Be Needed to Regulate New Food Technologies 26 (1993) (“Whereas FDA bears the burden of proving that a food product is adulterated after the product has entered the market, a manufacturer bears the burden of proving that a food additive’s use is safe before the additive may enter the market.”).

393
*See* Scrufari, *supra* note 59, at 235-43 (describing FDA’s lack of any meaningful pre- or post-market review).

394 Color Additive Petition from Center for Science in the Public Interest, et al., Request to Revoke Color Additive Listing for Use of FD&C Red No. 3 in Food and Ingested Drugs, 90 Fed. Reg. 4628, 4631 (Jan. 16, 2025) (to be codified at 21 C.F.R. pt. 74).

395
*See* Pomeranz et al., *supra* note 212, at 458-60.

396
*See* Neltner et al., *supra* note 157, at 88-90.

397 Doa, Fiksel & Maffini, *supra* note 322.

398
*See* 21 U.S.C. § 348(c)(5)(B). *See also* Soroudi, *supra* note 239, at 63-64 (advocating for “[c]omprehensive testing requirements, namely cumulative risk assessment,” for all GRAS determinations); Maffini & Neltner, *supra* note 371, at 496-99 (calling for an expanded cumulative risk assessment approach to evaluating the safety of additives).

399
*See* 21 U.S.C. § 360b(d)(2).

400
*See* Kate Schweitzer, *New Federal Program Seeks to Bridge Nutrition Research with Regulatory Policy*, 334 [J]AMA 103, 103-06 (June 6, 2025).

401
*Id.*

402 Doa, Fiksel & Maffini, *supra* note 322.

403
*See* 21 U.S.C. § 348(c)(5)(B).

404
*See* 21 C.F.R. § 610.1 (2025).

405
*Guidance for Industry: Action Levels for Poisonous or Deleterious Substances in Human Food and Animal Feed*, U.S. Food & Drug Admin. (Sep. 20, 2018), https://www.fda.gov/regulatory-information/search-fda-guidance-documents/guidance-industry-action-levels-poisonous-or-deleterious-substances-human-food-and-animal-feed [https://perma.cc/7BE3-4WH2].

406
*See, e.g.*, Joseph A. Levitt, *CFSAN’s Program Priorities: From Food Safety to Food Security*, 58 Food & Drug L.J. 19, 23 (2003) (discussing FDA’s agreements with states and other federal laboratories to avoid a strain on its resources due to increased testing); Jill Rosenthal, Hailey Gibbs & Allie Schneider, *5 Actions the FDA Can Take To Reduce Heavy Metal Toxins in Baby Food*, Ctr. for Am. Progress (Apr. 4, 2023), https://www.americanprogress.org/article/5-actions-the-fda-can-take-to-reduce-heavy-metal-toxins-in-baby-food/ [https://perma.cc/J3YV-4DKM] (discussing FDA’s limited resources for research and inspections of heavy metals).

407
*See* Alexander Tin, *FDA Making Plans to End Its Routine Food Safety Inspections*, *Sources Say*, CBS News (Apr. 18, 2025, at 11:55 ET), https://www.cbsnews.com/news/fda-food-safety-inspections-plans/ [https://perma.cc/96PT-2YGL].

408
*See* 21 C.F.R. § 211.84 (2025); 21 C.F.R. § 211.165 (2025).

409
*See* 21 C.F.R. § 101 (2025); *see also* 21 C.F.R. § 201.15 (2025) (listing criteria under which a drug’s labeling may lack the conspicuousness and prominence required by statute); 21 C.F.R. § 201.10 (2025) (listing FDA’s requirements for listing a drug’s ingredients on the label).

410 S.B. 25, 89^th^ Leg. (Tx. 2025).

411 Food Safety and Chemical Disclosure Act, S.B. 1239A, 2025-2026 S., Reg. Sess. (N.Y. 2025).

412
*Cf.* 21 C.F.R. § 201.57 (2025) (requiring all prescription drug labels to include, among other items, descriptions of data and studies supporting safe and effective use of the drug).

413
*See* Brejt, *supra* note 74, at 38.

